# *Schistosoma mansoni* egg-derived thioredoxin and Sm14 drive the development of IL-10 producing regulatory B cells

**DOI:** 10.1371/journal.pntd.0011344

**Published:** 2023-06-26

**Authors:** Mathilde A. M. Chayé, Thomas A. Gasan, Arifa Ozir-Fazalalikhan, Maaike R. Scheenstra, Anna Zawistowska-Deniziak, Oscar R. J. van Hengel, Max Gentenaar, Mikhael D. Manurung, Michael R. Harvey, Jeroen D. C. Codée, Fabrizio Chiodo, Anouk M. Heijke, Alicja Kalinowska, Angela van Diepen, Paul J. Hensbergen, Maria Yazdanbakhsh, Bruno Guigas, Cornelis H. Hokke, Hermelijn H. Smits

**Affiliations:** 1 Department of Parasitology, Leiden University Medical Center, Leiden, The Netherlands; 2 Department of Parasitology, Institute of Functional Biology and Ecology, Faculty of Biology, University of Warsaw, Warsaw, Poland; 3 Department of Immunology, Institute of Functional Biology and Ecology, Faculty of Biology, University of Warsaw, Warsaw, Poland; 4 Leiden Institute of Chemistry, Leiden University, Leiden, The Netherlands; 5 Italian National Research Council, Institute of Biomolecular Chemistry, Pozzuoli, Italy; 6 Witold Stefański Institute of Parasitology, Polish Academy of Sciences, Warsaw, Poland; 7 Museum and Institute of Zoology, Polish Academy of Sciences, Warsaw, Poland; 8 Center for Proteomics and Metabolomics, Leiden University Medical Center, Leiden, The Netherlands; NIAID-ICER, INDIA

## Abstract

During chronic schistosome infections, a complex regulatory network is induced to regulate the host immune system, in which IL-10-producing regulatory B (Breg) cells play a significant role. *Schistosoma mansoni* soluble egg antigens (SEA) are bound and internalized by B cells and induce both human and mouse IL-10 producing Breg cells. To identify Breg-inducing proteins in SEA, we fractionated SEA by size exclusion chromatography and found 6 fractions able to induce IL-10 production by B cells (out of 18) in the high, medium and low molecular weight (MW) range. The high MW fractions were rich in heavily glycosylated molecules, including multi-fucosylated proteins. Using SEA glycoproteins purified by affinity chromatography and synthetic glycans coupled to gold nanoparticles, we investigated the role of these glycan structures in inducing IL-10 production by B cells. Then, we performed proteomics analysis on active low MW fractions and identified a number of proteins with putative immunomodulatory properties, notably thioredoxin (SmTrx1) and the fatty acid binding protein Sm14. Subsequent splenic murine B cell stimulations and hock immunizations with recombinant SmTrx1 and Sm14 showed their ability to dose-dependently induce IL-10 production by B cells both *in vitro* and *in vivo*. Identification of unique Breg cells-inducing molecules may pave the way to innovative therapeutic strategies for inflammatory and auto-immune diseases.

## Author summary

Worm parasites, such as schistosomes, are master regulators of the human immune system, manipulating the host response in order to prolong their survival in their host. As a bystander effect, they also reduce immune responses to allergens and/or auto antigens, thus protecting their host against inflammatory and auto-immune diseases. One of the immune cells involved in immune regulation is the IL-10-producing regulatory B (Breg) cells. Schistosome eggs release various molecules that induce the development of Breg cells, but the identity of these molecules is not yet fully known. In this study, the authors aimed to identify some of these molecules. They investigated both the involvement of glycans on high molecular weight schistosomal egg molecules, as well as dissected the role of proteins with a lower molecular weight coming from schistosomal eggs. Using proteomics, they targeted various interesting molecules, which they recombinantly expressed and confirmed the IL-10 inducing capacity in B cells *in vitro* and *in vivo* for 2 molecules. This new knowledge may explain the hyporesponsiveness found in chronic schistosome-infected people and may pave the way to new innovative therapies for inflammatory and auto-immune diseases.

## Introduction

While B cells are known for their capacity to produce antibodies and induce protective immunity against invading pathogens, regulatory B (Breg) cells are part of a complex regulatory network of immune cells and can suppress inflammatory conditions by acting on antigen-presenting cells, such as dendritic cells or monocytes, or on effector T cells, such as Th1, Th2, Th17 and CD8+ T cells [1,2].

Various subsets of Breg cells have been distinguished, varying in context and suppressive mechanisms. In mice, CD21^hi^CD23^hi^ transitional 2-marginal zone precursors (T2-MZP) have been described in experimental arthritis [3], systemic lupus erythematosus (SLE) [4,5] and cancer [5]. CD1d^hi^CD5+ B10 Breg cells have been identified in the context of experimental autoimmune encephalomyelitis (EAE) [6], allergy [7], chronic colitis [8] and infections [9,10]. CD138+(LAG3+) plasma cells have also been observed in the context of autoimmunity [11], infections [12,13] and allergies [14], while TIM-1+ Breg cells have been described in graft tolerance [15], colitis [16] and in cancer [17,18].

Most Breg cells exert their function through the production of the anti-inflammatory cytokine IL-10 (reviewed in Rosser and Mauri, 2015 [2]), but depending on the subset, Breg cells can also produce the anti-inflammatory cytokines TGF-β [19] and/or IL-35 [20]. They can likewise exert their activity through the production of cytotoxic Granzyme B [21]. Furthermore, they can act in a cell-cell dependent manner, via their expression of FasL [22], PD-1 [23], PD-L1 [24] and/or CD1d [25].

Breg cell activity can be impaired in the context of autoimmunity, as shown in EAE [26], rheumatoid arthritis (RA) [27], SLE [28], chronic colitis [29], as well as in transplant rejection [30,31]. In contrast, Breg cells are overly active in other chronic conditions such as cancer where they are associated with disease progression [23,32,33]. Furthermore, Breg cells are increased in number and in activity in chronic infections with hepatitis C virus [34] or *Mycobacterium tuberculosis* [35]. Next to enhanced IgE and IgG1 antibody production against helminth antigens [36], increased development and activity of Breg cells is also found in helminth infections, and in particular infections with schistosomes. Previous findings in mice and humans show that helminth-induced Breg cells are able to affect CD4+ T cell response, reduce T cell proliferation, and induce Treg cells, thereby modulating immune responses in multiple sclerosis (MS) patients and protecting against allergic airway inflammation (AAI) [37–40]. Furthermore, isolated schistosomal eggs as well as *Schistosoma mansoni* soluble egg antigens (SEA) also induce Breg cells while avoiding the deleterious effects of live helminth infection [40–42]. Recently, IL-4 inducible protein (IPSE, a glycoprotein isolated from SEA) was identified as a *Schistosoma*-specific molecule, driving the development of IL-10-producing Breg cells [42]. Interestingly, SEA depleted from IPSE was still able to induce Breg cells to a similar extent, suggesting that other schistosomal egg-derived molecules have this activity as well [42]. In the present study, we aimed to identify new Breg-inducing egg-derived molecules from SEA. Using gel filtration size separation by Fast Protein Liquid Chromatography (FPLC), we identified both high and low molecular weight (MW) active fractions based on their ability to drive the development of murine IL-10 producing Breg cells *in vitro*. Using mass spectrometry, we next identified the components of the active low MW fractions and selected several candidate molecules for generation of recombinant molecules. Among them, we showed that *S*. *mansoni* thioredoxin-1 (SmTrx1) and Sm14 (a fatty acid binding protein), respectively obtained with HEK and yeast recombinant protein production systems, were both able to induce Breg cells *in vitro*.

Identification of new immunomodulatory molecules able to promote the development of Breg cells is of particular interest to explore novel therapeutic avenues for inflammatory and/or auto-immune diseases.

## Material and methods

### Ethics statement

All procedures involving animals were approved by the Animal Experiments Ethical Committee of the Leiden University Medical Center (AVD1160020173525). All animal experiments were performed in accordance with the Dutch Experiments on Animals Act is established under European Guidelines (EU directive no. 86/609/EEC regarding the Protection of Animals used for Experimental and Other Scientific Purposes).

### Animals

6–10 weeks old female C75Bl/6J OlaHsd mice were housed under specific-pathogen-free conditions in the animal facility of the Leiden University Medical Center in Leiden, The Netherlands.

### Hock immunization

Mice were injected subcutaneously into the hind hock with either PBS, 20 μg SEA, 20–50 μg SmTrx1 or Sm14 in 30 μL. Draining popliteal lymph nodes (LN) were analyzed 1 week later [43]. Cells were counted and stimulated with PMA (50 ng/mL) and ionomycin (1 μg/mL) in the presence of Brefeldin A (10 μg/mL) for 4 h. The cells were stained and analyzed as described below.

### Preparation of schistosome egg antigens

*S*. *mansoni* eggs were isolated from collagenase-digested hamster livers, 50 days post infection. Isolated eggs were washed in RPMI medium with 300 U/mL penicillin, 600 μg/mL streptomycin (Sigma-Aldrich) and kept at -80°C. SEA was prepared as previously described [44]. Antigen preparation was checked for endotoxin contamination (less than 40 ng/mg of protein) as tested by Limulus Amoebocyte Lysate (LAL) test.

### Gel filtration

4 mg of SEA was dialyzed against PBS with Slide-A-Lyzer dialysis cassettes (3.5K MWCO, Thermo Fisher Scientific, according to manufacturer’s protocol) and then fractionated using Sephacryl S300HR column (diameter 1 cm, length 20 cm) and AKTA Pure apparatus (GE Healthcare Life Sciences). SEA material was separated according to size at 4°C, using PBS at 0.3 mL/min. To compare the relative effect of different size fractions, a fixed volume of 25 μL of each SEA fraction was used to stimulate B cells.

### Protein concentrations

Protein concentrations in SEA fractions and recombinant molecules were determined by Bicinchoninic acid assay (BCA) (Pierce BCA Protein assay kit, Thermo Fisher Scientific) according to the manufacturer’s instructions.

### SDS-PAGE, silver staining, Coomassie staining and Western blot

Samples were separated by SDS-PAGE under reducing or non-reducing conditions using 12% acrylamide gels. Protein visualization was performed by silver staining, as previously described [45], or by Coomassie staining using Colloidal Blue Staining kit (Thermo Fisher Scientific) following manufacturer’s instructions. For Western blot, proteins were transferred onto PVDF membranes (GE Healthcare Life Science) and blocked overnight at 4°C in TBSTM buffer (Tris-buffered saline (TBS) with 0.1% Tween (Sigma-Aldrich) and 5% milk powder (Campina)). Membranes were incubated with primary antibody diluted in TBSTM for 1h at room temperature (RT), washed in TBSTM and incubated with the secondary antibody (rabbit polyclonal anti-mouse IgG-HRP, Dako), diluted 1:10,000 in TBSTM for 1h at RT. Monoclonal antibodies used for the detection of glycans include mAb 291-4D10-A (recognizes Lewis^X^ structures [46]), mAb 291-5D5-A (recognizes F-LDN(-F) structures [47]), mAb 114-4D12-AA (recognizes Fucα1-2Fucα1-R structures [48,49]). Detection was performed using Pierce ECL Plus Western blotting substrate (Thermo Fisher Scientific) and Super RX-N X-Ray films (Fujifilm), developed with the X-ray film processor (Huqju Imaging Technology).

### B cell isolation and stimulation

Single cell suspensions were obtained by dispersion of murine spleens through a 100 μm cell strainer (BD Biosciences), followed by erythrocytes lysis. B cells were purified from splenocytes using anti-CD19 microbeads (Miltenyi Biotec) following the manufacturer’s protocol. 3x10^5^ CD19+ B cells were cultured in 96-well round bottom plates (Sigma-Aldrich) in 200 μL RPMI 1640 Glutamax medium (Thermo Fisher Scientific) containing 5% heat-inactivated Fetal Calf Serum (FCS, Bodinco), 50 μM β-2-mercapthoethanol (Sigma-Aldrich), 100 U/mL penicillin and 100 μg/mL streptomycin (Sigma-Aldrich). Cells were stimulated with SEA (20 μg/mL), SEA fractions (25 μL), or recombinant molecules (SmTrx1, Sm14 or SmVAL28, 1–50 μg/mL) for a total of 48 hours. After 44 hours, supernatants were collected and kept at -20°C for later cytokine analysis by ELISA. Cells were restimulated during the last 4 hours with 100 ng/mL phorbol 12-myristate 13-acetate (PMA), 1 μg/mL ionomycin and 10 μg/mL Brefeldin A (Sigma-Aldrich) before flow cytometric analysis.

### Flow cytometry (FACS)

After staining for live/dead cells with Aqua dye (Thermo Fisher Scientific), mouse B cells were fixed with 1.9% paraformaldehyde and permeabilized using permeabilization buffer (eBioscience). B cells were then stained with fluorochrome-labelled antibodies against B220 (RA3-6B2, eBiosciences), IL-10 (JES5-16E3, eBiosciences) or CD86 (GL1, Biolegend). FcγR-binding inhibitor (2.4G2, Bioceros) was added to staining mixes and FMOs were used for gating. Reference samples on cells or beads were used for establishment of compensation matrix. Flow cytometry was performed on FACSCanto or LSR apparatus (BD Biosciences) and data was analyzed using FlowJo Software (BD Biosciences), version 10.7. Gating strategies are depicted in S1A Fig. For *in vivo* experiments, cells were treated as above and then stained with antibodies against CD25 (PC61, Biolegend), MHC II (2G9, BD Biosciences), CD44 (IM7, BD Biosciences), CD45 (30-F11, BD Biosciences), CTLA4 (UC10-4B9, Biolegend), IL-13 (eBio13A, eBiosciences), B220 (RA3-6B2, eBiosciences), CD80 (16-10A1, Biolegend), CD4 (RM4-5, Biolegend), CD8 (53–6.7, Biolegend), CD3 (17A2, Biolegend), PD1 (29f.1a12, Biolegend), IFNγ (XMG1.2, eBiosciences), IL-17 (eBio1757, eBiosciences), IL-10 (JES5-16E3, eBiosciences), FoxP3 (FJK-165, eBiosciences) and CD86 (GL1, Biolegend), together with FcγR-binding inhibitor (2.4G2, Bioceros) and diluted in permeabilization buffer and brilliant buffer (BD Biosciences). Samples were measured using Cytek Aurora spectral cytometer. Reference samples were used for unmixing using SpectroFlo software (Cytek) and FMOs were used for gating. Data was analyzed using FlowJo software (BD Biosciences), version 10.7. Gating strategy is depicted in S1B Fig.

### ELISA

The concentration of IL-6 and IL-10 present in culture supernatants was quantified by mouse ELISA kits according to the manufacturer’s instructions (BD Biosciences). Briefly, 96-well Nunc-Immuno polystyrene Maxisorp ELISA flat bottom plates (Thermo Fisher Scientific) were coated with Capture Antibody overnight at 4°C. After incubation, wells were washed with Wash Buffer and blocked with Assay Diluent. After subsequent washings, standards and samples were added to the wells. Wells were then washed, incubated with Working Detector (Detection Antibody + Streptavidin-horseradish peroxidase conjugate), and washed again. Substrate solution was then added to each well, plates were incubated 30 min in the dark before adding Stop Solution. Finally, absorbance at 450nm was read using a microplate reader.

### Chemical and enzymatic treatment of SEA fractions

#### Periodate treatment

One volume of cold 0.4 M acetate buffer (pH 4.5, Merck) containing 40 mM sodium metaperiodate (Merck) was added to one volume of SEA fractions. Samples were incubated overnight at 4°C while rotating. Quenching was performed by adding one volume of cold 50 mM sodium borohydride solution (Fluka) to the different samples and incubated for 30 min on ice. Treated SEA fractions were dialyzed against PBS using Slide-A-Lyzer MINI Dialysis Devices (3,5K MWCO, Thermo Fisher Scientific, according to the manufacturer’s instructions). Mock samples were subjected to the same treatment without the presence of sodium metaperiodate in the acetate buffer.

#### Trypsin treatment

To each of the SEA fractions 1.3% SDS and 1% β-2-mercaptoethanol was added and the samples were then incubated at 95°C for 10 min, before putting them on ice. Then NP-40 (Merck) was added to the samples to reach a final concentration of 1.3% NP-40. 40 μL of trypsin-coated Sepharose beads, prepared according to the manufacturer’s protocol (GE Healthcare) were added to each sample, which were then incubated overnight at 37°C while shaking at 250 rpm. Trypsin beads were removed by a series of centrifugation steps (400 rpm for 3 min), and potential trypsin released by the beads was deactivated by incubating samples for 10 min at 95°C. Finally, trypsin treated samples were dialyzed against PBS using Slide-A-Lyzer MINI Dialysis Devices (3,5K MWCO, according to manufacturer’s protocol).

### Generation of fucosylated gold nanoparticles

Mono-, di-, tri- and tetra-fucosylated 5nm gold nanoparticles were generated as described previously [49].

### Affinity chromatography

The monoclonal antibody 114-4D12-AA (mouse IgG1, here referred to as 114-4D12), which recognizes Fucα1-2Fucα1-R difucosyl motif) was coupled to Protein G Sepharose (Sigma-Aldrich) as described in Sisson and Castor, 1990 [50]. Next, 1 mg SEA in PBS and 1% sodium chloride was incubated with 114-4D12 coupled-beads (1 mg mAb) for 10 min before washing with 10 bead volumes (BV) of PBS. Then 114-4D12 reactive molecules were eluted with 5 BV of 0.1 M glycine-HCl solution, pH 1.5 (Sigma-Aldricht). The pH of elution fractions was neutralized with ammonium hydroxide solution (Fluka). Flow-through containing SEA depleted from 114-4D12 reactive molecules (SEAΔ114-4D12) as well as elution fractions were dialyzed against PBS using Slide-A-Lyzer MINI Dialysis Devices (3,5K MWCO, as per the manufacturer’s protocol).

Because of the apparent dense glycosylation of the affinity captured molecules, different volumes of elution fractions were used to stimulate B cells instead of protein concentrations. Splenic B cells were stimulated with 20 μg/mL SEAΔ114-4D12 and 5x this volume of elution fractions.

### Proteomics analysis

For sample clean-up, a short SDS-PAGE run of SEA fractions F12, F13 and F14 previously obtained by FPLC was performed. Gels were stained with SimplyBlue Safe Stain (Invitrogen) for 1 hour at RT and washed with distilled water for 3 hours. The full lane corresponding to each fraction was cut into four bands, and the proteins in each band were then subjected to reduction with dithiothreitol (10 mM), alkylation with iodoacetamide (50 mM) and in-gel digestion with trypsin (Worthington Enzymes), using a Proteineer DP digestion robot (Bruker). Tryptic peptides were extracted from the gel slices, lyophilized, dissolved in solvent A (95/3/0.1 water/acetonitrile/formic acid (FA) v/v/v), and subsequently analyzed by online C18 nano-HPLC MS/MS with a system consisting of an Easy nLC 1000 gradient HPLC system (Thermo Fisher Scientific) and a Orbitrap Fusion Lumos mass spectrometer (Thermo Fisher Scientific). Fractions were injected onto a homemade precolumn (100 μm × 15 mm; Reprosil-Pur C18-AQ 3 μm, Dr. Maisch, Ammerbuch, Germany) and eluted via a homemade analytical nano-HPLC column (15 cm × 50 μm; Reprosil-Pur C18-AQ 3 μm). The gradient was run from 10 to 40% solvent B (20/80/0.1 water/acetonitrile/FA v/v/v) in 30 min. The nano-HPLC column was drawn to a tip of ∼5 μm and acted as the electrospray needle of the MS source. The mass spectrometer was operated in data-dependent MS/MS (top-10 mode) with a normalized collision energy of 32% and recording of the MS2 spectrum in the Orbitrap. For peptide identification, MS/MS spectra were searched against the *S*. *mansoni* database (WormBase ParaSite, version 15.0, https://parasite.wormbase.org/Schistosoma_mansoni_prjea36577/, 14499 entries) in Proteome Discoverer 2.4 (Thermo), using Mascot Version 2.2.07 (Matrix Science). The following settings were used: 10 ppm and 20 milli mass units deviation for precursor and fragment masses, respectively; trypsin was set as the enzyme and two missed cleavages were allowed. Carbamidomethyl on cysteines was set as a fixed modification. Variable modifications were oxidation (on Met) and acetylation on the protein N-terminus. For the searches, the data from the four gel bands per sample (see above) were merged. Only proteins identified with at least two unique peptides were subsequently selected. The mass spectrometry proteomics data have been deposited to the ProteomeXchange Consortium via the PRIDE [51] partner repository with the dataset identifier PXD031333.

### Recombinant expression of *S*. *mansoni* thioredoxin and SmVAL28 in Exp293F cells

#### Cloning

Full-length, coding sequences (CDS) of SmTrx1 (Smp_008070.1) and SmVAL28 (Smp_176160) were obtained from Wormbase Parasite (parasite.wormbase.org). To prevent incorporation of mammalian glycans into the final protein, Asn-Gln substitutions were made at predicted sites of N-linked glycosylation (using NetNGlyc 1.0 (http://www.cbs.dtu.dk/services/NetNGlyc/)) at AA71 of SmTrx1 and AA6 of SmVAL28. Sequences were then codon-optimized for *Homo sapiens* prior to gene synthesis (GeneArt, Thermo Fisher Scientific). A C-terminal 6-His tag was incorporated into the target sequences for downstream purification. The SmTrx1 and SmVAL28 sequences were then cloned into the pSecTAG2A expression vector (Thermo Fisher Scientific) and sequenced by dideoxy chain-termination sequencing (LGTC sequencing facility, LUMC) to ensure in-frame insertion of correct sequences. The constructs were then transfected into Exp293F cells as per the manufacturer’s instructions for the Expi293F expression system (Gibco).

#### Protein expression and purification

Expi293F cells (Thermo Fisher Scientific) were cultured as described in the manufacturer guidelines. Cells were grown in Expi293F Expression Medium (Gibco) at 37°C 8% CO_2_, shaking at 125 rpm. Culture supernatants were collected 5 days after transfection and purified over a HisTrap Excel column (GE Healthcare) with 500 mM Imidazol (Merck) used for elution of bound proteins. Successful purification was determined by SDS-PAGE and Coomassie staining as described previously (S2A and S2B Fig). Elution fractions containing the pure target protein were buffer exchanged into PBS with PD10 desalting columns (GE Heathcare) as per manufacturer’s instructions. SmTrx1 and SmVAL28 identity was confirmed using in gel trypsin digestion and MALDI-TOF/MS. The endotoxin contamination (determined by LAL-assay) in the various batches of recombinant proteins is less than 40 ng/mg of protein in the stocks and ranges 0.05–0.3 ng/mL at the highest dose of the stimulation (20 ug/mL).

### Recombinant expression of *Schistosoma mansoni* Sm14 in *Pichia pastoris*

#### Cloning

The full-length CDS of Sm14 (Smp_095360) was obtained from Wormbase Parasite (parasite.wormbase.org) and amplified from cDNA by PCR. Amplicons were cloned into pGEM-T Easy vectors (Promega) via T/A cloning and then, subcloned into the yeast expression vector pPICZαC with His-tag sequence (Invitrogen) using *Cla*I and *Xba*I restriction sites (underlined). Primer sequences were as follows:

F-ClaI-Sm14 GCCATCGATCATGTCTAGTTTCTTGGGAAAGTG
R-XbaI-Sm14 GCCTCTAGACAGGATAGTCGTTTATAATTGCG.

Inverse PCR was used to remove N-glycosylation site predicted with NetNGlyc 1.0 (http://www.cbs.dtu.dk/services/NetNGlyc/): codon encoding asparagine residue 59 was changed into glutamine. Correct sequence of recombinant plasmid was confirmed by nucleotide sequencing. Recombinant plasmid was then linearized with *PmeI* and transformed into Pichia pastoris X33 strain using electroporation method.

#### Protein expression and purification

Recombinant Sm14 with His-tag was expressed by induction with 0.5% methanol for 72 h in Buffered Methanol-complex Medium (1% yeast extract (Sigma Aldricht), 2% peptone (Biocorp), 100 mM potassium phosphate, pH6 (Merck), 1.34% YNB (Biocorp), 4 x 10^−5^% biotin (AppliChem), 0.5% methanol (Merck)), and then purified from culture media using Ni-NTA resin columns (Macherey-Nagel) and NPI-250 elution buffer (50 mM NaH_2_PO_4_, 300 mM NaCl, 250 mM imidazole (Merck), pH 8). Eluted fractions were then concentrated and dialyzed against PBS using the AMICON system (Merck). In the next step, endotoxins were removed using Endotoxin Removal Spin Columns (Pierce) and filtered using 0.22 μm syringe filter. The purified recombinant Sm14 was analyzed by SDS-PAGE. Glycoprotein Staining Kit (Pierce) was used to confirm removal of glycan moieties. S2C and S2D Fig shows the results of Sm14 purification. The endotoxin contamination (determined by LAL-assay) in recombinant yeast Sm14 stock solution is and 0.03 ng/ml at the highest dose of the stimulation (50 μg/mL).

### Statistical analysis

#### Effect of trypsin and periodate treatment on SEA fractions

To estimate the magnitude of interaction between sample MW (high, medium, or low) and treatment, we fitted a linear mixed model (lme4 R package ver. 1.1–26) with interaction term between treatment and sample MW with random intercept for mice and sample ID. P-values were obtained by semi-parametric bootstrap with 1,000 iterations as implemented in parameters R package (version 0.13.0). Analysis was conducted using R version 4.0.4 in Rstudio version. 1.4.1103.

#### Others

All data is presented as mean ± standard error of the mean (SEM). Statistical analysis was performed with GraphPad Prism version 7 for Windows (Graph Pad software, La Jolla, CA, USE) using either one-way ANOVA followed by Dunnett’s multiple comparisons tests or Kruskal-Wallis with Dunn’s multiple comparisons test (for normalized GeoMFI). All p-values below 0.05 were considered significant and are represented on figures by * p < 0.05, ** p < 0.01, *** p < 0.001.

### Structural representation of glycans

Glycan structures were drawn with GlycoWorkbench 1.0 software [52].

## Results

### High, medium and low molecular weight fractions of SEA drive IL-10 production by B cells

**Fig 1:**
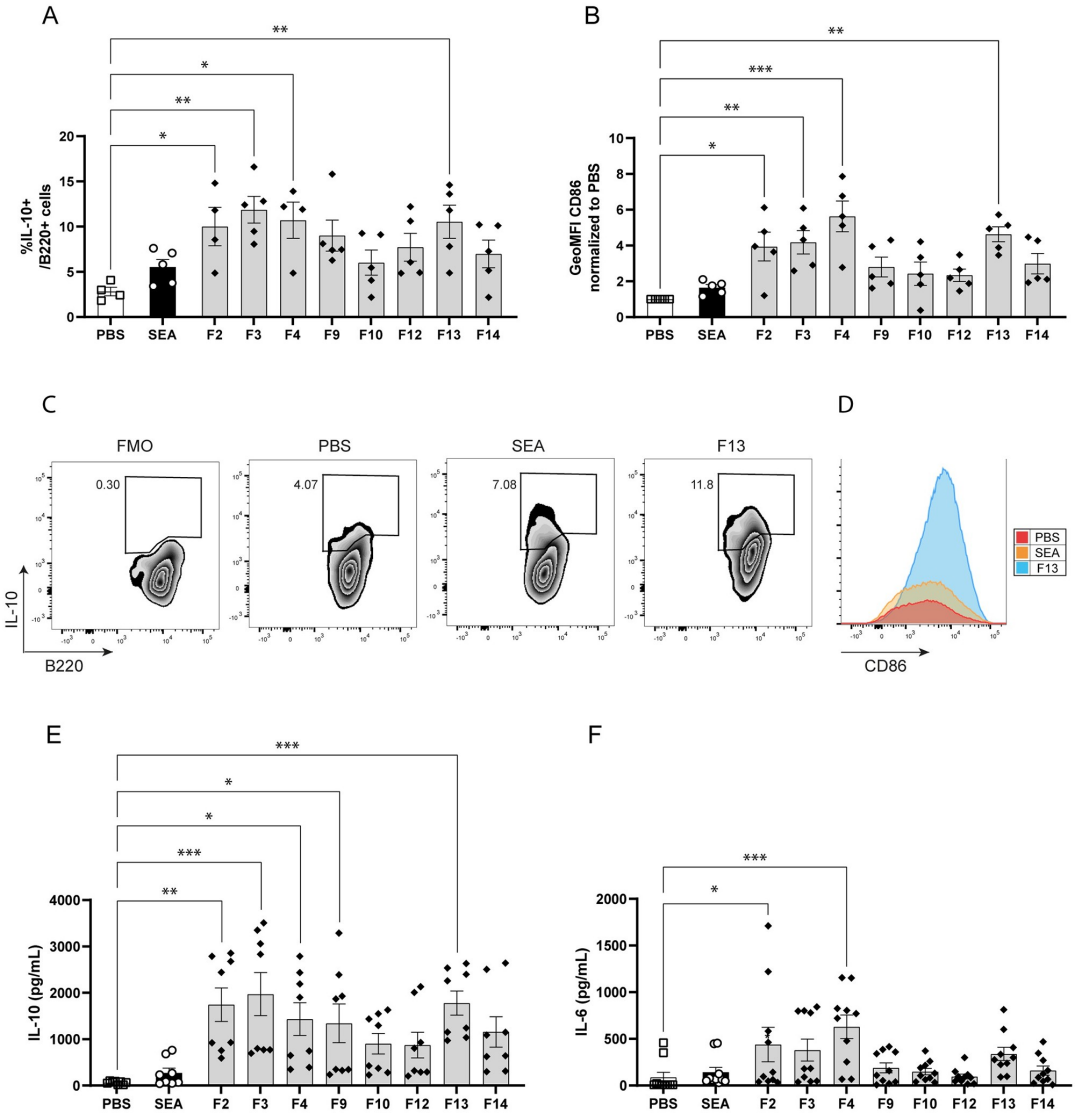
Molecular size-specific SEA fractions show a differential capacity to induce IL-10 production in B cells. Splenic CD19^+^ cells were isolated from naïve mice by anti-CD19 microbeads and stimulated (1.5x10^6^ cells/mL) with different molecular size fractions of SEA (generated by gel filtration with Sephacryl S300HR column) for 48 hours, while PMA, ionomycin and Brefeldin A were added for the last 4 hours. Intracellular IL-10 production (A) and CD86 Geometric Mean Fluorescence Intensity (GeoMFI) (B) were analyzed by flow cytometry. Representative FACS plots for intracellular IL-10 (C) and extracellular CD86 (D) expression of B cells for FMO (staining control), PBS, SEA and F13 are shown. Secreted IL-10 (E) and IL-6 (F) in supernatant were measured by ELISA. n = 5 with fractions from one representative gel filtration experiment out of two. Dotted lines separate the high, medium and low molecular weight fractions. Error bars represent SEM, * p<0.05, ** p<0.01, *** p<0.001, compared to PBS condition. One-way ANOVA with Dunnett’s multiple comparisons test were performed to determine statistical significance, except for CD86 GeoMFI, which was normalized to PBS and then analyzed using Kruskal-Wallis with Dunn’s multiple comparisons test.

Previously, we identified the glycoprotein IPSE/alpha-1 secreted by *S*. *mansoni* eggs as a Breg-inducing molecule [42]. Interestingly, SEA depleted from IPSE could still induce IL-10 production by murine splenic cells. In order to gain more knowledge on other putative Breg-inducing molecules in SEA, the antigen mixture was fractionated by size exclusion chromatography into 18 fractions (S3A and S3B Fig). Isolated murine splenic B cells were stimulated with the obtained fractions. After 48h, B cells stimulated with fractions containing relatively high molecular weight (MW) components (F2 to F4), medium MW components (F9 and F10), or with low MW material (F13 and F14) showed a significant or a trend towards an increase in the number of cells expressing the anti-inflammatory cytokine IL-10 and/or the activation marker CD86, compared to B cells stimulated with other fractions (Fig 1A–1D). Furthermore, these cells showed increased production of IL-10 while keeping a relative low expression of inflammatory cytokine IL-6 (Fig 1E and 1F and S3C and S3D Fig). As the stimulations were performed with equal volumes of the fractions, rather than similar protein levels, this may have affected the outcome in the IL-10 inducing activity. Therefore, we have normalized the IL-10 and IL-6 levels for protein content in each fraction (S3E and S3F Fig). Interestingly, after normalization by protein content the low MW fractions F13 and F14 and the high MW fraction F2 showed the strongest B cell IL-10 inducing activity, as well as enhanced IL-6 production. However, the increased IL-10 induction observed in medium and other high MW fractions was not visible anymore, presumable because these fractions were more protein rich. One caveat, if activity is linked to other molecules, such as glycans or lipids, this finding would be masked if we would rely on protein-corrected activity only.

As this IL-10-inducing activity is observed in distinct SEA fractions (i.e., with high, medium or low MW molecules), this suggest that different and multiple molecules could be responsible for the IL-10-inducing and/or the IL-6 inducing activity of SEA. SEA is a complex mixture consisting of thousands of different molecules, in particular proteins and glycoproteins [42,48,53,54]. From previous studies, we know that the high MW substances in SEA are heavily glycosylated and that glycans can play a role in the induction of IL-10 in B cells [55], therefore we investigated if glycans could play a role in driving Breg cell development.

### Role of SEA-containing heavily glycosylated molecules in Breg cells development

**Fig 2:**
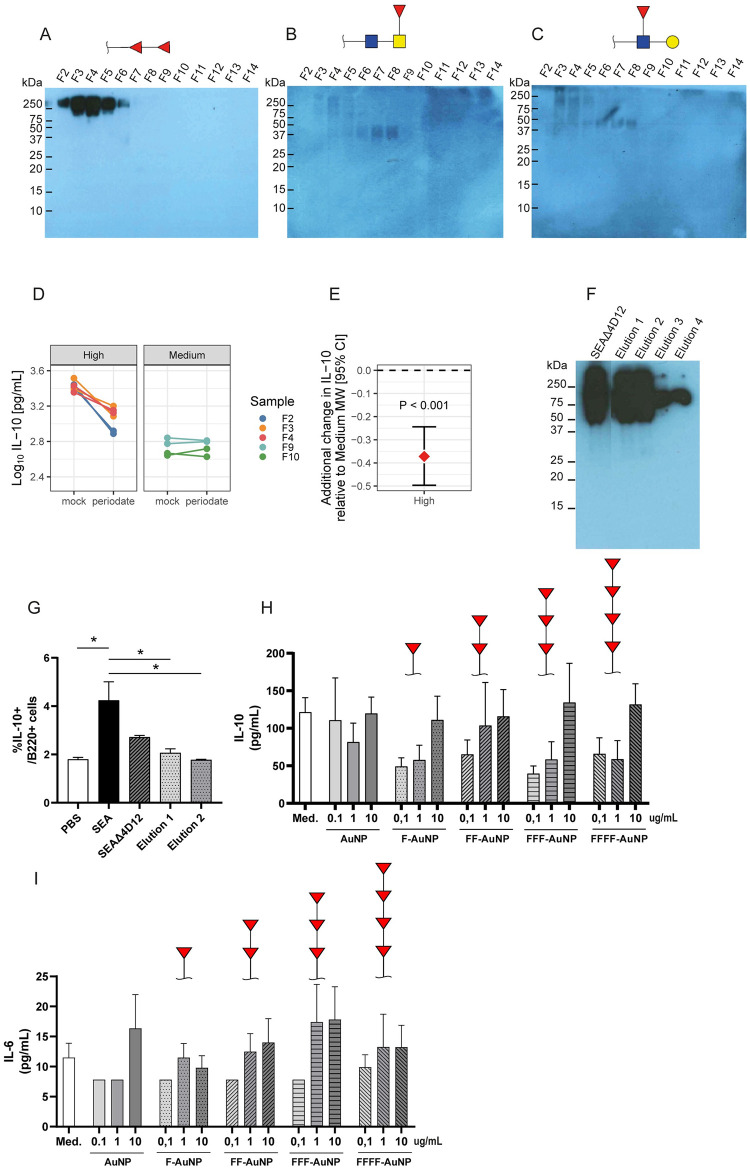
Highly glycosylated substances in high MW SEA fractions contribute to IL-10 production in B cells. (A-C) Different molecular size fractions of SEA (generated by gel filtration with Sephacryl S300HR) were run on a 12% reducing SDS-PAGE gel and blotted on PVDF membranes. Blots were incubated with a specific monoclonal antibody (mAb) against double fucosylated residues (FF, 114-4D12 mAb) (A), fucosylated LacdiNAc (F-LDN, 291-5D5 mAb) (B) or Lewis X (LeX, 291-4D10 mAb) (C). Glycan motifs recognized are depicted above each blot (structures designed using GlycoWorkbench). (D) Glycans present in the different molecular size fractions of SEA were oxidized using periodate treatment (overnight incubation at 4°C with 40mM sodium metaperiodate in 0.4M acetate buffer (pH4.5), followed by 30min incubation with 50mM sodium borohydride on ice. Samples were dialyzed using Slide-A-Lyzer MINI Dialysis Devices (3.5K MWCO). Effect of periodate treatment was compared to that of mock treated samples (samples underwent the same treatment without sodium metaperiodate). Subsequently, these treated fractions were used to stimulate splenic B cells from naïve mice, as described to the legend of Fig 1A. IL-10 secretion in supernatant was measured by ELISA. Differential effect of treatment of different MW fractions on IL-10 production by B cells was estimated using linear mixed model. n = 2, exact p-values and estimate (95% CI) are depicted in S1 Table. (E) Estimated additional change (95% CI) in IL-10 production by B cells stimulated with periodate treated-high MW fractions, compared to periodate treated-medium MW fractions and relative to mock treatment. IL-10 secretion in supernatant was measured by ELISA. n = 2, exact p-values and estimate (95% CI) are depicted in S2 Table. (F) SEA was depleted from glycosylated molecules harboring the fucosylated motifs recognized by mAb 114-4D12 using affinity chromatography. Following incubation of 114-4D12 coupled-beads with SEA and subsequent washings, 114-4D12 reactive molecules were eluted using a glycine-HCl buffer solution and dialyzed against PBS. Elution fractions 1–4 correspond to the 2^nd^ until the 5^th^ mL of elution buffer applied on the affinity column and are the elution fractions containing the 114-4D12 bound molecules. SEA depleted from 114-4D12 bound molecules (SEAΔ4D12) and elution fractions were run on a 12% reducing SDS-PAGE gel, blotted on PVDF membrane and a western blot was performed with mAb 114-4D12. (G) Splenic CD19+ cells from naive mice were stimulated with SEA, SEAΔ4D12 and the elution fractions and analyzed by flowcytometry, as described in the legend for Fig 1. n = 2. (H-I) Splenic B cells from naïve mice were isolated and stimulated by various concentrations of empty, mono-, di-, tri- or quatri-fucosylated gold nanoparticles [49], as described in the legend to Fig 1 (n = 2). IL-10 (H) and IL-6 (I) secretion in supernatant was measured by ELISA. Glycan motifs present on the AuNP are depicted above each condition. Error bars represent SEM, * p < 0.05, ** p < 0.01, *** p < 0.001, compared to PBS condition. One-way ANOVA with Dunnett’s multiple comparisons test were performed to determine statistical significance.

Immunostaining using antibodies against glycans that contain the Fucα1-2Fucα1-R difucosyl motif (FF, Fig 2A), fucosylated LacDiNac (F-LDN, Fig 2B) or Lewis^X^ (Fig 2C) showed the presence of glycosylated high MW molecules in fractions F2 to F4, but not in medium (F9 and F10) or low (F12 to F14) MW fractions. In particular, fractions F2 to F4 were strongly recognized by the FF-reactive monoclonal antibody (mAb) 114-4D12 compared to antibodies against F-LDN or Lewis^X^, indicating the abundant presence of difucosylated motifs in the high MW molecules. Previous studies have shown that such difucosyl motifs bound by mAb 114-4D12 often are part of larger multi-fucosylated structures [48,49,56].To investigate if these glycan structures are involved in SEA-induced Breg cell development, high and medium MW fractions were either mock treated or treated with sodium metaperiodate in order to oxidize glycans and prevent their recognition by lectin receptors [57–60]. Fig 2D and 2E and S1 and S2 Tables show that periodate treatment significantly reduced the ability of high MW fractions to induce IL-10 production by B cells (p < 0.001), but not that of medium MW fractions (p = 0.89).

As glycosylated molecules in high MW fractions rich in multi-fucosylated glycans seem to play a role in IL-10 induction in splenic B cells, we next aimed to separate these multi-fucosylated molecules from the other SEA components using mAb 114-4D12 affinity chromatography. Fig 2F shows that mAb 114-4D12 bound glycoconjugates were successfully and strongly enriched in the elution fractions, although a significant amount of fucosylated molecules were still present in the remainder of the SEA (labeled SEAΔ4D12). Subsequent stimulation of splenic B cells showed that the IL-10 induction by SEAΔ4D12 was significantly lower compared to SEA. However, none of the 114-4D12 elution fractions were able to induce IL-10 production by splenic B cells (Fig 2G). As the affinity chromatography procedure may have affected the activity of the glycosylated substances and may have failed to select an active subset of fucosylated molecules, we decided to explore an alternative approach to study the role of glycosylated molecules present in SEA in Breg cell development, by using glycans coupled to gold nanoparticles (AuNP). The presence and bio-recognition by mAbs of these glycans on the AuNP was confirmed as previously reported [49]. We applied simple representations of fucosylated glycan motifs present in SEA glycoproteins [48,56] to generate fucosylated AuNP, including the Fucα1-2Fucα1-R difucosyl motif that is recognized by mAb 114-4D12. While simplistic in their nature compared to the complex glycans and glycoconjugates in SEA, these fucosylated AuNP do allow a controlled comparison of the effect of small defined glycan elements. Moreover, by coupling the glycans to AuNP a multivalent presentation of the fucosides is achieved, similar as during presentation on complex branched glycans and in highly glycosylated proteins. Multivalence is an important determinant in many glycan-lectin interactions [61,62]. Therefore, despite their simplistic nature, the synthetic fucoses coupled to AuNP form an excellent tool to study the role of single or multimeric fucoses in Breg cell development. Murine splenic B cells were stimulated with AuNP without glycans or coupled to mono-, di-, tri- or tetrafucoses. We observed a trend to a dose-dependent effect in the ability to induce IL-10 production in B cells by fucosylated AuNP, but there was no statistical difference compared to the spontaneous IL-10 secretion in unstimulated cells or in cells stimulated with AuNP without glycans (Fig 2H). These results suggest that such simple fucose motifs, normally present at the termini of larger complex glycoconjugates in SEA [48]–in an isolated setting–are not sufficient to induce IL-10 production in splenic B cells.

Our attempts to study the role of fucosylated substances in SEA, using affinity chromatography and synthetic glycans did not lead to conclusive results regarding the direct and unique involvement of the heavily fucosylated molecules, as present in high MW fractions in SEA, in the induction of Breg cells development. As such their role in Breg cell development remains open.

### Identification of schistosome proteins able to induce Breg cells in the low MW fractions of SEA

**Fig 3:**
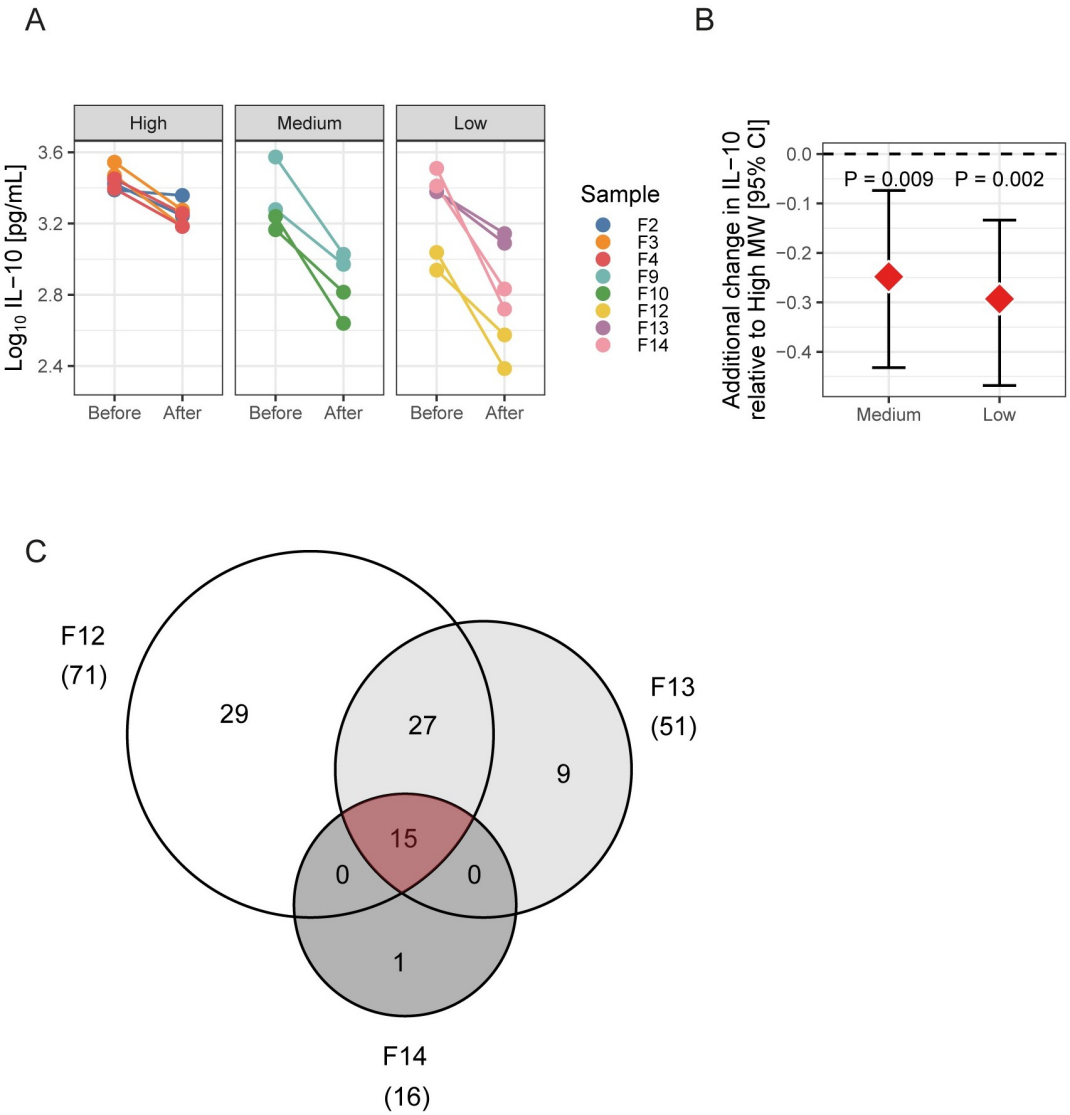
In low MW SEA fractions, proteins significatively promote IL-10 induction in B cells. (A) High, medium and low MW fractions of SEA were treated with trypsin (10 min incubation at 95°C in 1.3% SDS + 1% β-2-mercaptoethanol, followed by overnight incubation with trypsin beads and 1.3% NP-40 at 37°C and 250 rpm. Samples were cleaned by multiple centrifugation steps, 10 min incubation at 95°C and dialysis against PBS with Slide-A-Lyzer MINI Dialysis Devices (3.5K MWCO)). Subsequently, these fractions, before and after trypsin treatment, were used to stimulate splenic B cells from naïve mice, as described to the legend of Fig 1. IL-10 secretion in supernatant was measured by ELISA. n = 2, exact p-values and estimate (95% CI) are depicted in S3 Table. (B) Estimated additional change (95% CI) in IL-10 production by B cells stimulated with trypsin treated-low or -medium MW fractions, compared to high MW fractions. IL-10 production was determined by ELISA. Statistical modeling was performed using linear mixed model. n = 2, exact p-values and estimate (95% CI) are depicted in S4 Table. (C) Venn diagram representing the number of unique and common proteins identified by mass spectrometry (LC-MS/MS) in the low molecular weight fractions F12, F13 and F14.

Next, we studied the possible Breg-inducing potential of schistosome egg proteins. Trypsin is a serine protease that catalyzes the hydrolysis of peptide bonds, breaking down proteins into smaller peptides. Therefore, by trypsinizing the gel filtrated fractions of SEA, we could examine the role of the primary peptide sequence on Breg induction. As shown in Fig 3A and 3B, treatment of fractions with trypsin significantly altered their capacity to induce IL-10 production in splenic B cells (p = 0.003 for high MW, < 0.001 for medium and low MW fractions). The effect of the treatment was significantly higher in fractions from medium or low MW compared to high MW (p = 0.009 for medium MW and 0.002 for low MW fractions). Interestingly, even though the protein content of low MW fractions was less than half of the amount found in medium MW fractions (S3B Fig), these fractions were nevertheless more potent to induce IL-10 production by splenic B cells (Fig 1A and 1C). This suggests not only a strong activity in the low MW fractions of SEA, but also a less diverse composition and therefore increased chances for a successful identification of active proteins by mass spectrometry. Therefore, we focused on low MW fractions for the remaining analysis and the fractions F12, F13 and F14 were processed by LC-MS/MS. As shown in Fig 3C, in total 81 proteins were identified by searching the MS/MS spectra against the *S*. *mansoni* database WormBase ParaSite (proteins presented in S5–S7 Tables), among which 15 were present in all three fractions (area in red in Fig 3C). These 15 proteins are depicted in Table 1, sorted according to the Peptide-Spectrum-Matches (PSM) of fraction F13, the most active of low MW fractions. Based on their estimated function, molecules with a household or mitochondrial function were excluded from further analysis. Based on previous literature (see Table 1) and their relative abundance in active fractions (an arbitrary cut-off was set at a PSM value of 20 in individual fractions), we chose to focus on SmTrx1, Sm14 and SmVAL28. Of note, we had difficulties expressing Cyclophilin B and excluded it here from the analysis. We cannot exclude it may also have IL-10 inducing activity. Interestingly, IPSE, a known Breg cells inducer we previously reported on [42], was on this list, although ranked in the lowly abundant proteins.

**Table 1:** Identification of proteins present in SEA low MW fractions. Common proteins identified by LC-MS/MS in low MW fractions F12, F13 and F14, sorted according to Peptide-Spectrum-Matches (PSM) of fraction F13. Only proteins for which at least 2 unique peptides were detected are listed.

Protein ID	Smp ID	Description	PSM		
			F12	F13	F14
Ubiquitin (Ribosomal protein L40), putative	Smp_046690.2	Household protein	70	112	56
Thioredoxin	Smp_008070.1	Putative immunoregulatory molecule [74]	40	94	16
Venom allergen-like (VAL) 28 protein	Smp_154260.1	Putative immunoregulatory molecule [96]	54	88	40
Venom allergen-like (VAL) 27 protein	Smp_154290.1	Putative immunoregulatory molecule [96]	42	72	39
Cytochrome c-like protein; Putative cytochrome c	Smp_033400.2	Household protein	32	52	21
Peptidyl-prolyl cis-trans isomerase B (Cyclophilin B)	Smp_040790.1	Anti-viral vaccine candidate, cyclophilin family play a role in regulation of inflammatory responses [97,98]	63	45	7
14 kDa fatty acid-binding protein (Sm14)	Smp_095360.1	Anti-helminth vaccine candidate, putative immunoregulatory molecule [83,84,88]	49	42	13
Thioredoxin (Mitochondrial), Trx M	Smp_037530.1	Mitochondrial protein	18	29	13
Unknown	Smp_320540.1	Unknown	6	13	2
Peptidyl-prolyl isomerase	Smp_079230.1	Cyclophilin family play a role in regulation of inflammatory responses [98]	6	12	8
Unknown	Smp_336250.1	Unknown	62	11	2
Peptidyl-prolyl cis-trans isomerase (Cyclophilin A)	Smp_040130.1	Putative immunoregulatory molecule, cyclophilin family play a role in regulation of inflammatory responses [67,68,98]	11	9	15
Protein disulfide-isomerase	Smp_079770.1	Thioredoxin domain	12	6	3
IL-4-inducing protein (IPSE)	Smp_112110.1	Known Breg cells inducer [42,44]	10	5	2
Kunitz-type protease inhibitor, putative	Smp_052230.1	Anti-schistosoma vaccine candidate [99]	9	5	3

### SmTrx1 and Sm14 promote IL-10 production by B cells *in vitro*

**Fig 4:**
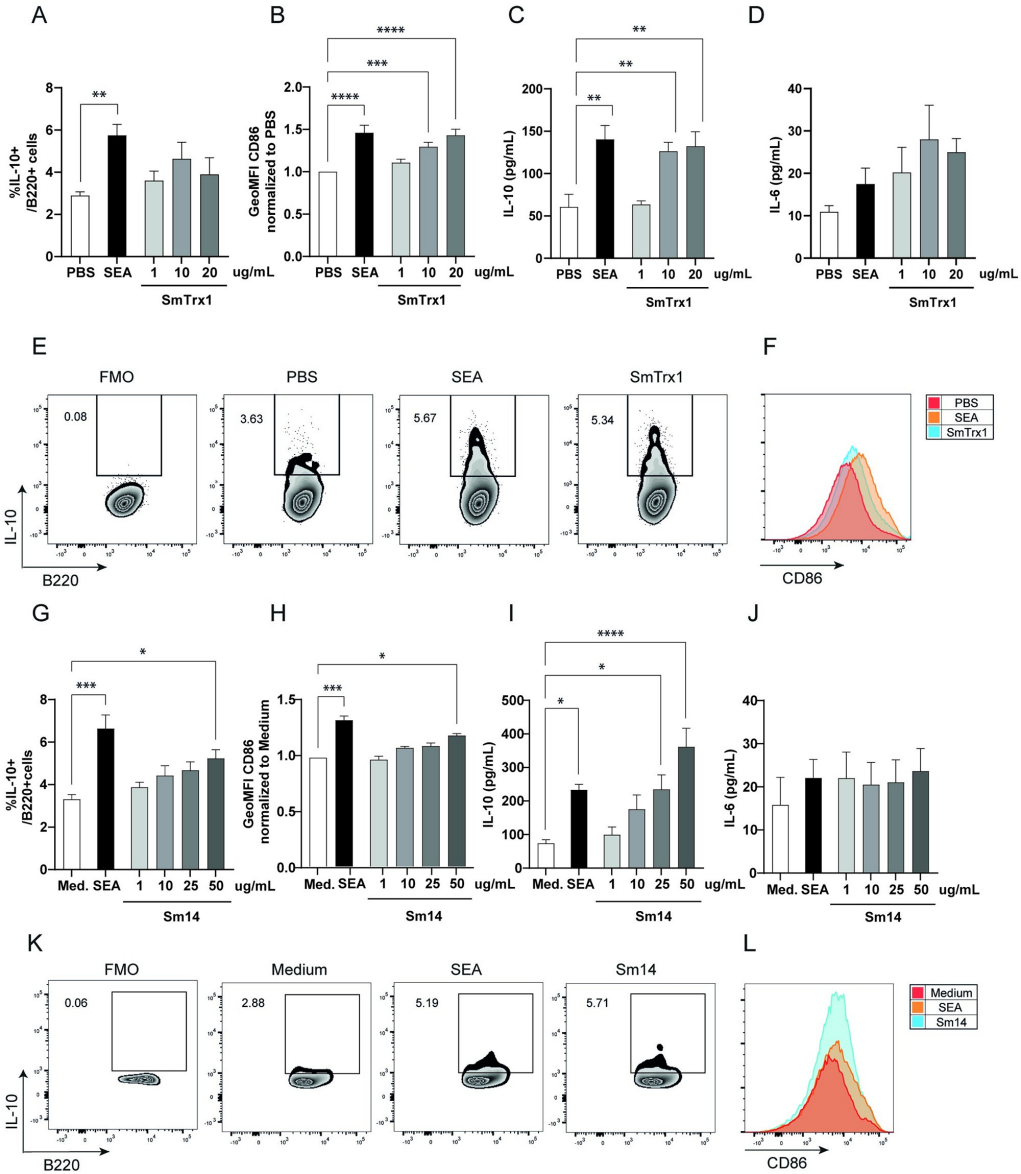
Recombinant SmTrx1 and Sm14 induce IL-10 production in splenic B cells. His-tagged SmTrx1 and Sm14 were recombinantly expressed in Exp293F cells and in *Pichia pastoris* yeast, respectively. Following purification by affinity chromatography, recombinant SmTrx1 and Sm14 were used in the splenic B cell assay, according to the legend to Fig 1A. Intracellular IL-10 production (A; G) and CD86 GeoMFI (B; H) were assessed by flowcytometry, while IL-10 (C; I) and IL-6 (D; J) secretion in supernatant were measured by ELISA. Representative FACS plots for intracellular IL-10 (E; K) and CD86 (F; L) expression of B cells for IL-10 FMO, PBS, SEA, SmTrx1 and Sm14 are shown. n = 10–13 experiments for SmTrx1, using 4 independent batches. n = 5 for Sm14, using 1 batch. Error bars represent SEM, * p < 0.05, ** p < 0.01, *** p < 0.001, compared to PBS or medium condition. One-way ANOVA with Dunnett’s multiple comparisons test were performed to determine statistical significance, except for CD86 GeoMFI, which was normalized to PBS or medium and then analyzed using Kruskal-Wallis with Dunn’s multiple comparisons test.

SmTrx1, Sm14 and SmVAL28 were recombinantly expressed and tested for their ability to induce IL-10 production in splenic B cells. As shown in Fig 4A–4D, recombinant SmTrx1 was able to induce CD86 upregulation (a marker of B cell activation) and IL-10 production in a dose-dependent manner, but without the induction of the pro-inflammatory IL-6. However, no clear increase in the percentage of IL-10+ B cells were detected by intracellular flow cytometry. Of note, for SmTrx1 we observed a discrepancy between ELISA and FACS results, which has been reported in the context of induction of Breg cells with Toll-like receptors [63]. This difference is likely due to the distinct nature of the assays and may arise as a consequence of restimulation with PMA, ionomycin and Brefeldin A (intracellular FACS) versus no restimulation (ELISA) of the B cells.

Similarly, recombinant Sm14 was able to induce increased expression of CD86 and IL-10 production in a dose dependent manner, without affecting IL-6 secretion (Fig 4E–4H). Furthermore, B cells stimulated with 50 ug/mL recombinant Sm14 had an increased proportion of IL-10+ B cells at 48h (Fig 4E). Contrary to SmTrx1 and Sm14, SmVAL28 is not able to induce increased CD86 or IL-10 expression by B cells (S4 Fig).

**Fig 5:**
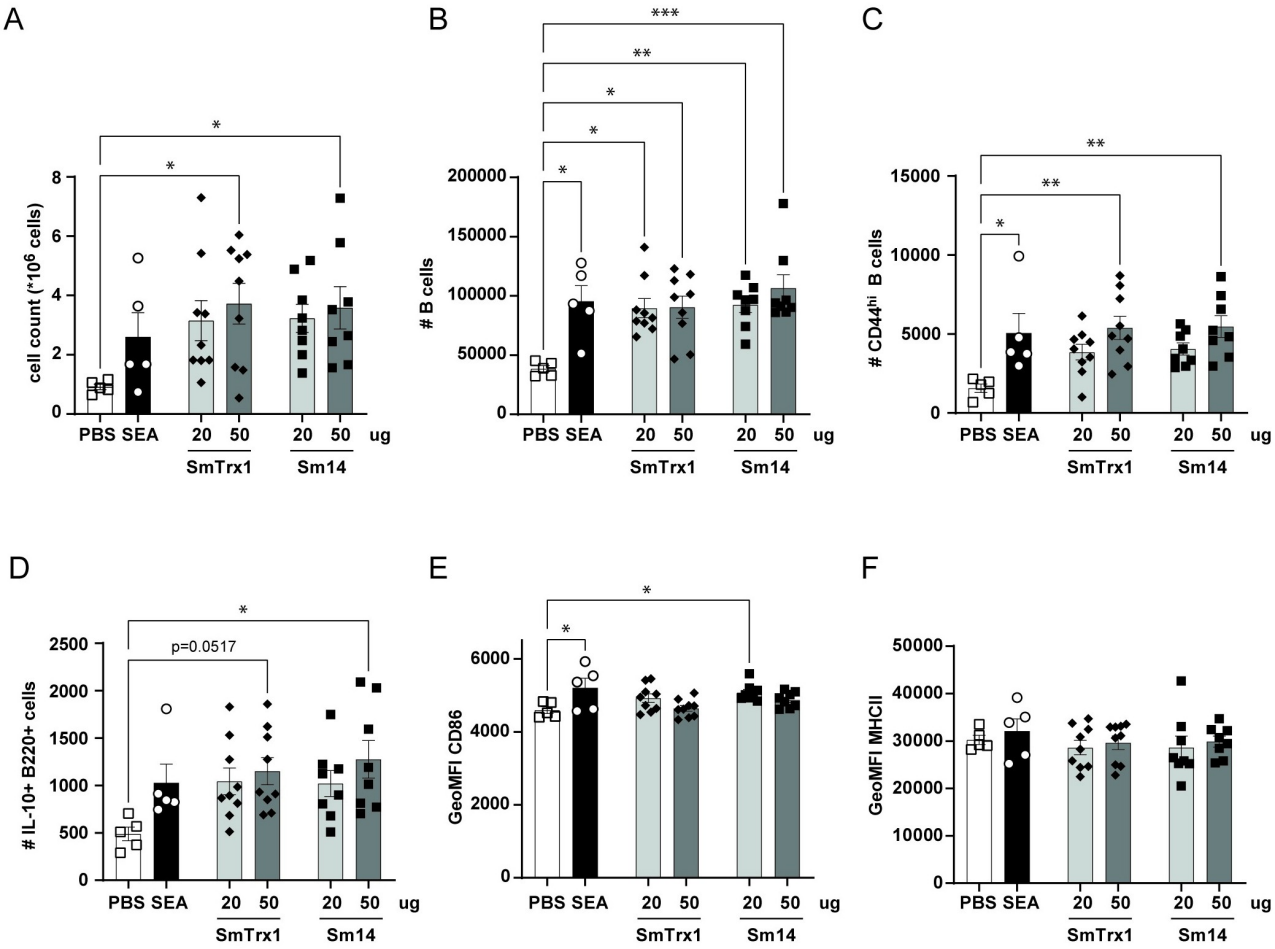
B cell responses following hock immunization with SmTrx1 and Sm14. Mice were immunized s.c. into hock with PBS, 20 μg SEA, 20 or 50 μg SmTrx-1 or Sm14, and the draining popliteal LNs were analyzed 1 week later. Cells were counted (A) and stimulated with PIB for 4 h. B220+ B cells (B), CD44hi B cells (C), IL-10+ B cells (D) and CD86 and MHCII GeoMFI (E and F) were analyzed by flow cytometry and frequencies were calculated on a per cell basis. n = 2. Error bars represent SEM, * p < 0.05, ** p < 0.01, *** p < 0.001, compared to PBS condition, with one-way ANOVA with Dunnett’s multiple comparisons test.

Next, we injected SmTrx1 and Sm14 (with SEA as control) in the hock of naïve mice and isolated single cells from the popliteal lymph nodes (LN) after 7 days. We observed a significant increased number of immune cells in the lymph nodes in response to the highest dose of SmTrx1 and Sm14 (Fig 5A), as well as an increase in the number of (CD44^hi^) B cells and (total, CD4+ and CD8+) T cells for SEA, SmTrx1 and Sm14 (at both 20 and 50 μg) immunized hock compared to PBS conditions (Fig 5B and 5D and S5A–S5C Fig). Following PMA, ionomycin and Brefeldin A restimulation of the LN cells and intracellular cytokine staining, we observed a significant increase in IL-10 producing B cells with the highest dose of Sm14 and to a lesser extent for the highest dose of SmTrx1 (p = 0.0517; Fig 5C). Costimulatory molecules were not so much affected: only an increase in GeoMFI of CD86 for SEA and Sm14 (lowest dose) (Fig 5E), while MHCII levels were not affected by any of the injected molecules (Fig 5F). Furthermore, FoxP3+ CD25+ regulatory T (Treg) cell numbers were more abundant in mice injected with the lowest dose of SmTrx1 and Sm14 (S5D Fig), while the number of CTLA4+ CD4 T cells was increased in SEA, SmTrx1 and Sm14 treated mice (both doses) (S5E Fig). As for cytokine production in CD4 T cells, we observed more cells producing IFNγ with SmTrx1 (lowest dose; S5F Fig), more IL-10+ CD4 T cells in response to SmTrx1 (both doses) and to Sm14 (highest dose: S5G Fig), while CD4 T cell numbers producing IL-17+ were not affected (S5H Fig). In summary, the (CD4) T cells mostly seem to develop towards more regulatory responses (Treg phenotype and cytokines) in line as what is observed for the IL-10 producing B cells.

All together, these findings show that recombinant SmTrx1 and Sm14 are able to induce IL-10 production in splenic B cells *in vitro* and in popliteal B cells *in vivo*, suggesting that these schistosome-derived molecules have the capacity to drive to development of murine IL-10 producing Breg cells.

## Discussion

Schistosome parasites and schistosome eggs are strong inducers of IL-10 producing Breg cells. The main goal of this study was to identify novel Breg-inducing molecules in SEA. After identifying active high and low MW IL-10 inducing B cell fractions in SEA, we investigated a putative dominant role of schistosomal glycans in high MW fractions of SEA and demonstrated the capacities of SmTrx1 and Sm14, identified in the active low MW fractions, to induce IL-10 production by B cells.

*Schistosoma* glycans are unusual and distinct from human glycans: in SEA there are no sialic acids, which are abundant on human glycoproteins, but instead contains many fucoses (mono-, di- or trifucoses residues as epitopes), LDN and Lewis^X^ motives [56]. Interestingly, earlier studies have suggested a role for specific SEA glycans in IL-10 induction in B cells [64]. Indeed, oxidation of glycans by periodate treatment in active high MW SEA fractions also suggested a role for helminth glycans here. However, our attempts to identify the dominant motif were unsuccessful, maybe because enrichment by affinity chromatography was not optimal, even though SEA-depleted from fucosylated molecules (using mAb 114-4D12) was less active than non-depleted SEA. There is a possibility that the procedure affected the structures of the glycans, also altering their activity and/or binding properties [65]. A subsequent attempt to study the role of complex glycans in SEA by gold-nanoparticles coupled to synthetic fucose oligomers showed that these very simple structures were not able to drive IL-10 production in B cells. As the synthetic structures did not reach the same complexity as the native SEA-contained fucosylated glycoconjugates, it remains an open question whether these same structures in the full context of the glycosylated branches in SEA and/or in the presence of other SEA molecules/structures with complementary activity would provide the capacity to drive Breg cell development.

As mentioned before, the concept that schistosomal glycans can induce IL-10 production by B cells has first been described by Velupillai and Harn in 1994 who showed that splenic B cells stimulated with lacto-N-fucopentaose III (LNFPIII), a polylactosamine from SEA, produce IL-10 [64]. Interestingly, LNFPIII contains a Lewis^X^ motif which can also be found in the 2 most abundant glycoproteins of SEA, IPSE and omega-1. IPSE is able to drive Breg cells development, but omega-1 cannot [42]. While omega-1 cellular activity is dependent on binding to the lectin receptor DC-SIGN and MR through Lewis^X^ in dendritic cells (DCs) to condition them for Th2 priming, for IPSE this seems not to be essential for uptake by DCs [66] nor for its induction of IL-10 in B cells, as demonstrated in SIGNR1-deficient mice [42].

After our investigation into the role of glycans in SEA high MW fractions, we also studied the role of proteins in the low MW fractions. Following LC-MS/MS identification, 15 proteins were present in all 3 fractions of interest. Among them, the presence of known Breg cell inducer IPSE should be noted [42,44], as well as that of previously described Cyclophilin A [67,68]. However, these proteins were present only in low abundance in the low MW fractions, making it less likely that they play a dominant role in driving B cell IL-10 production and Breg cells development from these low MW fractions. We had technical difficulty to express Cyclophilin B, so we cannot exclude a role of Cyclophilin B in IL-10 induction in this stage. We identified two abundant SEA proteins with Breg inducing potential: SmTrx1 and Sm14, most probably actively secreted by live *S*. *mansoni* eggs [69,70]. Even if no previous studies demonstrated or hinted at a possible role in Breg cell development for either protein, the combination of their relative abundance in active SEA fractions as well as the description of their activity in different contexts lead us to investigate their activity on B cells. Another abundant protein in the active low MW fractions, SmVAL28, did not show the IL-10-inducing capacity in B cells.

Trx1 is a small cytosolic and extracellular 12 kDa protein with a characteristic Cys-Gly-Pro-Cys site which confers its protein disulfide reductase activity. Together with thioredoxin reductase (TrxR) and NADPH, Trx1 is part of the thioredoxin system, the cell’s most important redox regulation component. Through its redox activity on other proteins such as ribonucleotide reductase, methionine sulfoxide reductase or Trx1-dependant peroxidases, Trx1 play a critical role in DNA synthesis, protein repairs and protection against oxidative stress. It also affects the activity of many transcription factors such as NF-κB, p53, Ref-1, HIFα, PTEN, AP-1, or glucocorticoid receptor, consequently acting on apoptosis, transcription, cell cycle arrest and redox signaling [71,72]. Trx1 also acts as a chemoattractant for neutrophils, monocytes and T cells [73], but most importantly Trx1 was able to promote the polarization of macrophages into anti-inflammatory M2 macrophages and to reduce the LPS-induced differentiation into inflammatory M1 macrophages, both *in vitro* and *in vivo*. In a mouse model of atherosclerosis, treatment with Trx1 led to decreased numbers of M1 macrophages and reduced atherosclerotic lesions [74].

These findings were made using human Trx1, which is 46.7% identical to the *S*. *mansoni* SmTrx1 and possesses the same protein disulfate activity site [75,76], but clearly thioredoxins from other origins can have an immunomodulatory role as well. For example, *Leishmania infantum* tryparedoxin (LiTXN1), which belongs to the superfamily of thioredoxins, can drive B cell activation, proliferation, and IL-10 secretion *in vitro* [77]. With this study, we are the first to demonstrate that *S*. *mansoni* SmTrx1 exhibits a capacity to induce IL-10 production in B cells. Interestingly, SmTrx1 is 47% identical to human Trx1 at the amino acid sequence level and displays the same characteristic redox site.

Finally, we report that the fatty acid binding protein (FABP) Sm14 was also able to drive the production of IL-10 by B cells *in vitro*. FABPs are small 14–15 kDa intracellular proteins that bind lipids with high affinity. Mammalian FABPs are a collection of 9 homologues, highly conserved through tissues and species. Their main function is to coordinate lipid responses inside cells, by facilitating fatty acids solubilization, trafficking and metabolism, but also by interacting with various membrane and intracellular proteins, such as peroxisome proliferator-activated receptors (PPARs) or hormone sensitive lipase (HSL) (reviewed in Storch and Corsico, 2008 [78]). FABPs have also been described in several helminth species, like *Fasciola hepatica* [79], *Echinococcus spp* [80], *Brugia malayi* [81], and *Schistosoma mansoni* [82]. FABPs are of particular interest as these parasites are unable to synthesize lipids *de novo*, relying on carriers such as FABPs to take up lipids from the host [82]. For this reason, Sm14 is an interesting antihelminthic drug target, and a Sm14/GLA-SE vaccine has entered human clinical trials [83]. Phase 1 clinical trial data showed specific IgG response and no IgE, as well as the production of a mixture of Th1 and Th2 cytokines, including the anti-inflammatory IL-10 [84]. This is consistent with previous work on *S*. *mansoni* infection resistant individuals and vaccination studies in mice [85,86]. These cytokines are part of the ‘normal’ response to worms and eggs and protect the host against serious consequences of continued production of inflammatory mediators, as part of the chronic infection. Interestingly, previous studies in PBMCs of *S*. *mansoni* infected individuals showed increased IL-10 production in response to Sm14, although the cellular source was not studied [87].

Several studies have reported an immunomodulatory role for *F*. *hepatica* FABP, which is 49% identical to *S*. *mansoni* FABP Sm14 with a conserved fatty acid binding domain: both native and recombinant *F*. *hepatica* FABPs can induce the alternative activation of macrophages, but they also downregulate LPS-induced secretion of inflammatory cytokines TNFα, IL-12α and IL-1β, and stimulate IL-10 expression [88]. Additionally, *F*. *hepatica* FABP can suppress inflammatory responses and skew them towards a Th2 response in a mice model of EAE [89]. Interestingly, the activity of *F*. *hepatica* and *S*. *mansoni* FABP seems largely overlapping as vaccination of mice by Sm14 equally protects against *S*. *mansoni* and against *F*. *hepatica* infection, suggesting that their immunomodulatory effect may also be similar [90].

Using a mouse model of hock immunization, we tested the activity of SmTrx1 and Sm14 *in vivo*, and compared it to PBS injections. While we could see increased B cell numbers in general and IL-10 producing B cells in particular in popliteal lymph nodes immunized with Sm14 and SmTrx1 (a trend towards significance for SmTrx1 (p = 0.0517)) (Fig 5), B cells were not the only cells responding to the molecules. Indeed, we also observed more T cells, both CD4+ and CD8+, of FoxP3+ CD25+ Treg cells as well as changes in the profile of cytokine producing-T cells (S5 Fig). This effect is most likely the consequence of a direct interaction of SmTrx1 and Sm14 with other antigen presenting cells, like DCs, or a downstream effect of these molecules through the intermediary of B cells. Indeed, previous studies using human Trx1 and F. hepatica FABP, with similar sequence and active sites to our SmTrx1 and Sm14, have been shown to interact with macrophages and DCs, respectively, leading to more anti-inflammatory macrophages, more IL-10-producing DCs and downregulation of LPS stimulating effects [74,89]. Therefore, it would be interesting to investigate in future studies the separate roles of other immune cells responding to SmTrx1 and Sm14, as well as to test the molecules’ effect in disease models.

While we used a standardized protocol to stimulate murine B cells with helminth recombinant molecules without adjuvants, cytokines or co-stimulation, we cannot rule out (the lack of) differences in IL-10 production in the additional presence of those factors [91]. Furthermore, the IL-10 production levels observed here may not be comparable to those found in response to bacterial or viral PAMPs (such as TLR-7, -8 or -9 ligands); however, those ligands also induce substantial levels of pro-inflammatory cytokines, such as IL-6, which may affect their regulatory potential.

Additionally, our study was limited to IL-10 producing B cells, as model for Breg cells, while we have not investigated the induction of other Breg cell cytokines or effector molecules, like IL-35, TGF-β or Granzyme B, or specific surface molecules which may have a regulatory function, such as PD-L1 or FasL [92]. Future studies should address the development of other potential Breg cell subsets. Likewise, it remains to be investigated whether SmTrx1 or Sm14 affect other B cell functions and/or (protective) antibody production as observed in (mouse models of) helminth infections [93–95].

## Conclusion

To conclude, we identified SmTrx1 and Sm14 as potential immunomodulatory molecules from *S*. *mansoni* able to stimulate the development of IL-10 producing Breg cells, both *in vitro* and *in vivo*. Breg cells are important players in the maintenance of immune homeostasis and dysregulations in Breg cells numbers and/or function are associated with a variety of pathologies such as auto-immune diseases, chronic infections, or cancers. While further research is needed to understand how SmTrx1 and Sm14 act on B cells and if the effects observed *in vitro* and through hock immunizations can be reproduced in disease models, these findings are promising in the development of new therapies for immune-related pathologies.

## Supporting information

S1 Fig Gating strategies. (A) Gating strategy for the identification of IL-10^+^ B220^+^ B cells. (B) Gating strategy for hock immunization experiments and the identification of total B220^+^ B cells, IL-10^+^ B cells, CD44^+^ B cells, CD4^+^ and CD8^+^ T cells, FoxP3^+^CD25^+^, CTLA-4^+^, IFNγ^+^, IL-10^+^ and IL-17^+^ CD4 T cells.
(TIF)

S2 Fig Expression and purification of recombinant SmTrx1 and Sm14. SmTrx1 and SmVAL28 were expressed with His-tag in Exp293F cells and purified on HisTrap Excel column. Analysis by SDS-PAGE on 10% agarose gel and staining with Coomassie showed the presence of SmTrx1 band (A) and SmVAL28 band (B) around the expected weight. Sm14 was expressed with His-tag in *Pichia pastoris* X33 strain and purified from culture media through Ni-NTA resin column. SDS-PAGE analysis on 10% agarose gel and Coomassie staining (C) revealed presence of hyper glycosylated form of Sm14 molecule, which resulted in change of expected molecule size and multiple bands presence (line 1). Next, potential N-glycosylation site was removed by modification of asparagine residue 59 into glutamine, and purified molecule was present as one band with expected size (line 2). Staining with Glycoprotein Staining kit (D) confirmed the presence and absence of hyperglycosylated forms of Sm14 before (line 1) and after (line 2) modification of Asn59. Line 3 and 4 showed negative and positive controls, respectively. M: weight marker.
(TIF)

S3 Fig Protein profile of SEA fractions. Different molecular size fractions of SEA (generated by gel filtration on Sephacryl S300HR column) were run on a 12% agarose SDS-PAGE and stained with silver staining (A). (B) Protein concentration of SEA fractions was measured using BCA. Figure shows one representative BCA measurement of one gel filtration experiment.
(TIF)

S4 Fig Recombinant SmVAL28 does not induce IL-10 production in splenic B cells. His-tagged SmVAL28 was recombinantly expressed in Exp293F cells, purified by affinity chromatography, and used in the splenic B cell assay, according to the legend to Fig 1A (n = 2). Intracellular IL-10 production (A) and CD86 GeoMFI (B) were assessed by flow cytometry. Representative FACS plots for intracellular IL-10 (C) and CD86 (D) expression of B cells for IL-10 FMO, PBS, SEA and SmVAL28 are shown. Secretion of IL-10 (E) and IL-6 (F) in supernatant were measured by ELISA.
(TIF)

S5 Fig T cell responses following hock immunization with SmTrx1 and Sm14. Mice were immunized s.c. into hock with PBS, 20 μg SEA, 20 or 50 μg SmTrx-1 or Sm14, and the draining popliteal LNs were analyzed 1 week later. Cells were counted, stimulated with PIB for 4 h and analyzed by flow cytometry. Figure shows the cell numbers of total T cells (A), CD4+ T cells (B), CD8+ T cells (C), FoxP3+CD25+ CD4 T cells (D), CTLA4+ CD4 T cells (E) as well as IFNγ, IL-10 and IL-17 producing CD4 T cells (F-H). n = 2. Error bars represent SEM, * p < 0.05, ** p < 0.01, *** p < 0.001, compared to PBS condition, with one-way ANOVA with Dunnett’s multiple comparisons test.
(TIF)

S1 Table Estimated change in IL-10 production by B cells after stimulation with periodate treated-high or -medium MW fractions, relative to mock treatment. (TIF)

S2 Table Additional change in IL-10 production by B cells stimulated with periodate treated-high MW fractions, relative to mock treatment, compared to medium MW fractions. (TIF)

S3 Table Estimated change in IL-10 production by B cells after stimulation with trypsin treated-high, -medium or -low MW fractions. (TIF)

S4 Table Additional change in IL-10 production by B cells stimulated with trypsin treated-low or -medium MW fractions compared to high MW fractions. (TIF)

S5 Table List of proteins identified in fraction F12 by LC-MS/MS, with more than 2 unique peptides, sorted according to Peptide-Spectrum-Matches (PSM). (TIF)

S6 Table List of proteins identified in fraction F13 by LC-MS/MS, with more than 2 unique peptides, sorted according to Peptide-Spectrum-Matches (PSM). (TIF)

S7 Table List of proteins identified in fraction F14 by LC-MS/MS, with more than 2 unique peptides, sorted according to Peptide-Spectrum-Matches (PSM). (TIF)
