# Supplementary figures and images for: Schistosoma mansoni egg-derived thioredoxin and Sm14 drive the development of IL-10 producing regulatory B cells
Source: PLoS Negl Trop Dis. 2023 Jun 26;17(6):e0011344. doi: 10.1371/journal.pntd.0011344 (PMC10328244; doi:10.1371/journal.pntd.0011344)

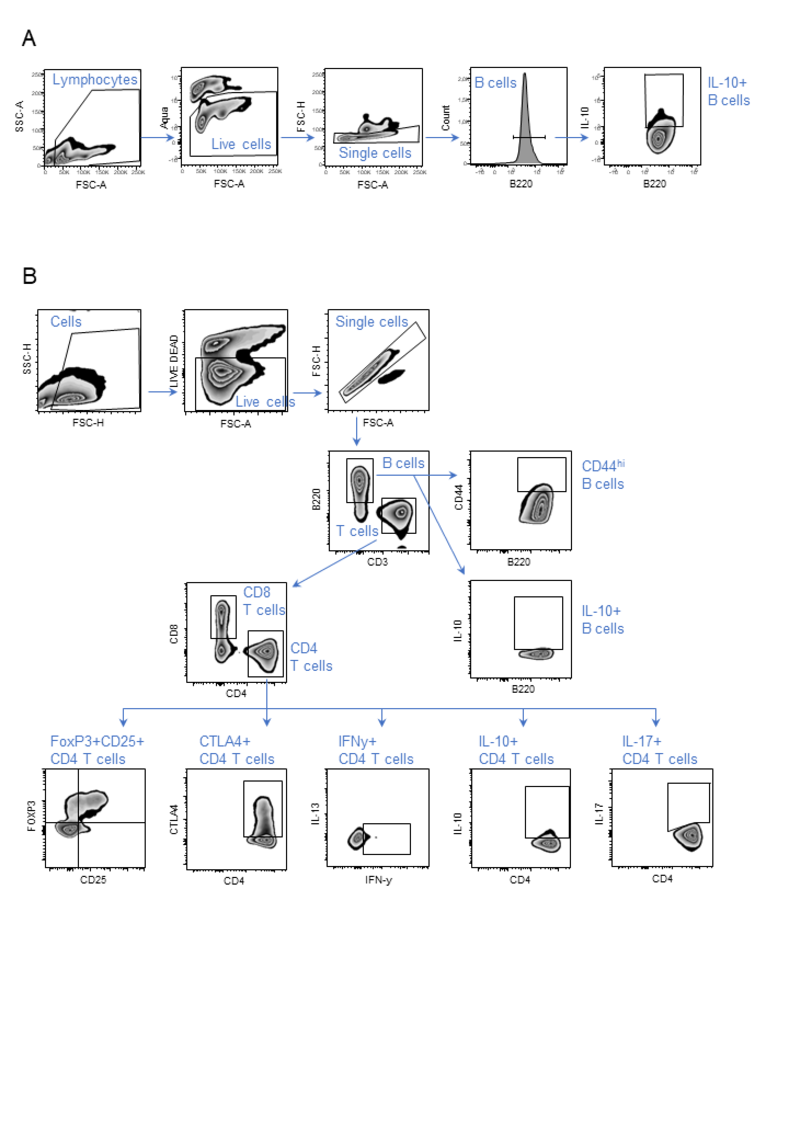

Supplement: S1 Fig — (A) Gating strategy for the identification of IL-10+ B220+ B cells. (B) Gating strategy for hock immunization experiments and the identification of total B220+ B cells, IL-10+ B cells, CD44+ B cells, CD4+ and CD8+ T cells, FoxP3+CD25+, CTLA-4+, IFNγ+, IL-10+ and IL-17+ CD4 T cells. (TIF) [file pntd.0011344.s001.tif]

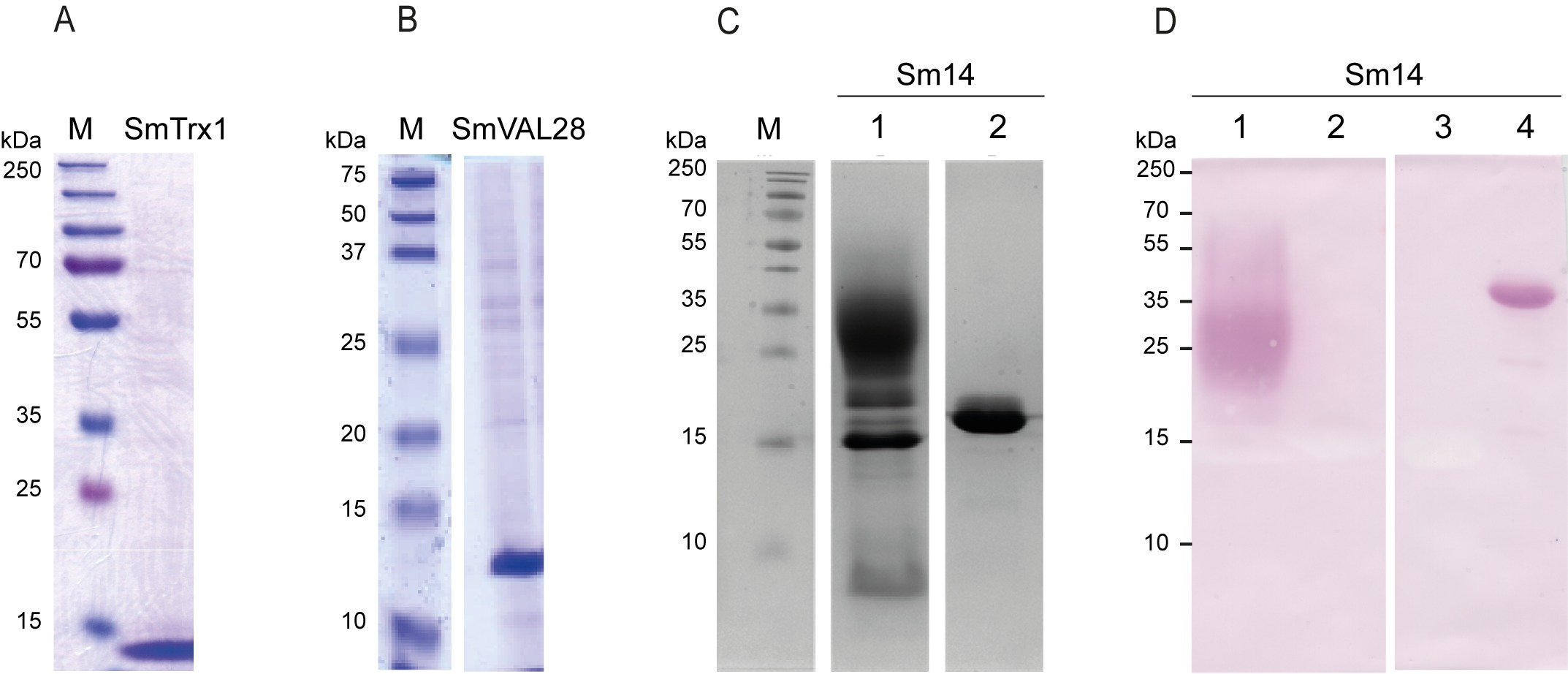

Supplement: S2 Fig — SmTrx1 and SmVAL28 were expressed with His-tag in Exp293F cells and purified on HisTrap Excel column. Analysis by SDS-PAGE on 10% agarose gel and staining with Coomassie showed the presence of SmTrx1 band (A) and SmVAL28 band (B) around the expected weight. Sm14 was expressed with His-tag in Pichia pastoris X33 strain and purified from culture media through Ni-NTA resin column. SDS-PAGE analysis on 10% agarose gel and Coomassie staining (C) revealed presence of hyper glycosylated form of Sm14 molecule, which resulted in change of expected molecule size and multiple bands presence (line 1). Next, potential N-glycosylation site was removed by modification of asparagine residue 59 into glutamine, and purified molecule was present as one band with expected size (line 2). Staining with Glycoprotein Staining kit (D) confirmed the presence and absence of hyperglycosylated forms of Sm14 before (line 1) and after (line 2) modification of Asn59. Line 3 and 4 showed negative and positive controls, respectively. M: weight marker. (TIF) [file pntd.0011344.s002.tif]

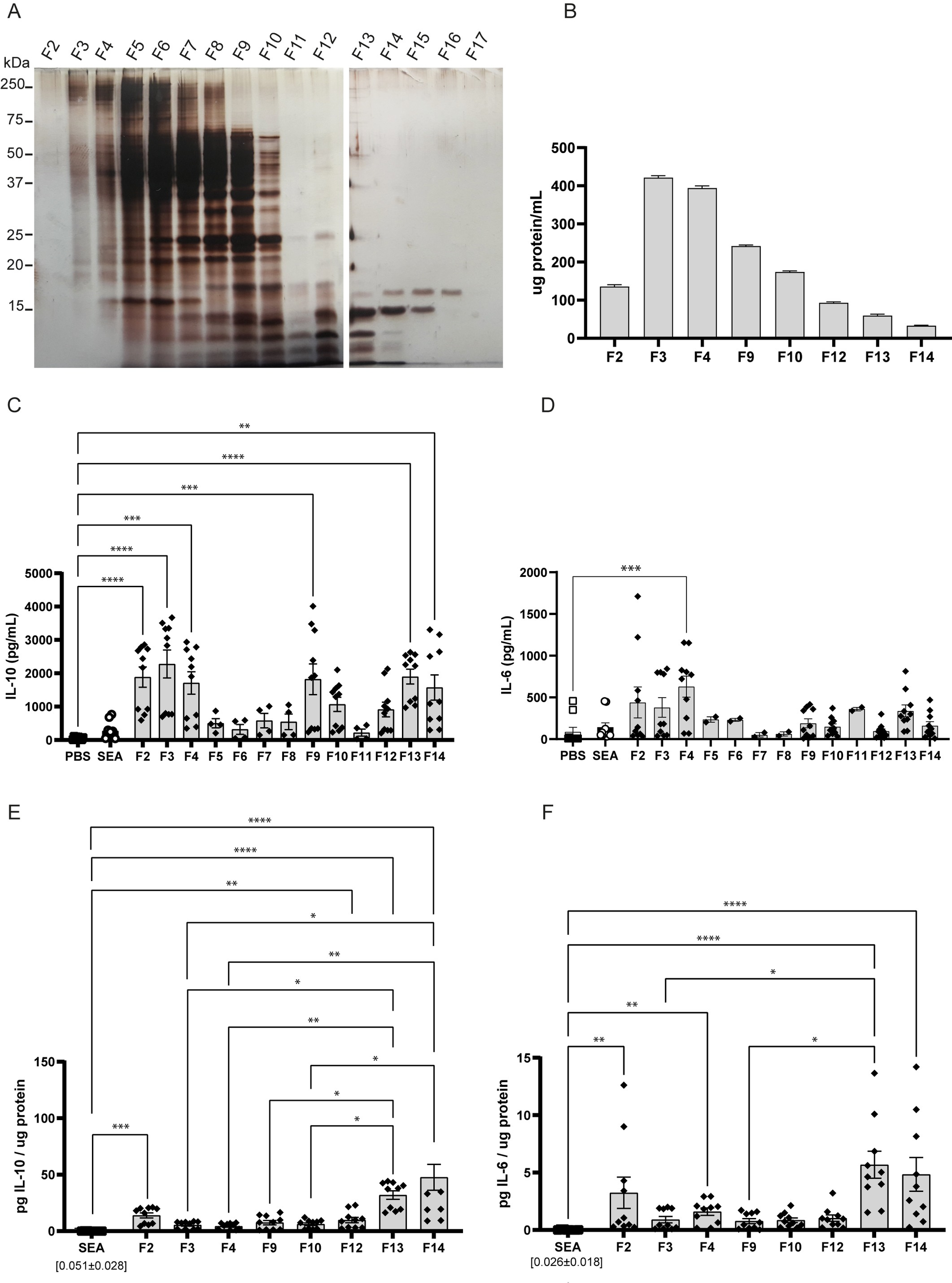

Supplement: S3 Fig — Different molecular size fractions of SEA (generated by gel filtration on Sephacryl S300HR column) were run on a 12% agarose SDS-PAGE and stained with silver staining (A). (B) Protein concentration of SEA fractions was measured using BCA. Figure shows one representative BCA measurement of one gel filtration experiment. (TIF) [file pntd.0011344.s003.tif]

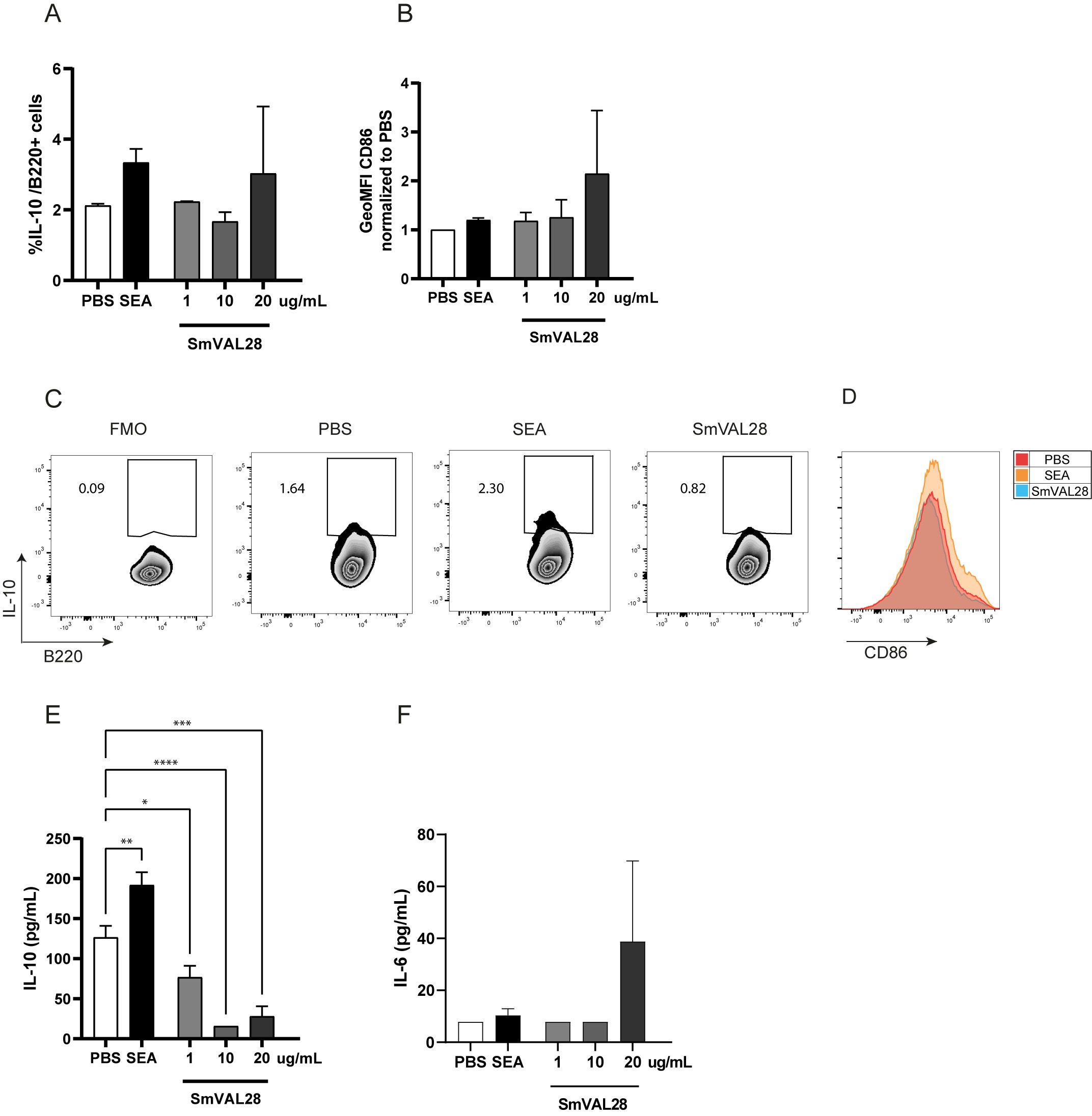

Supplement: S4 Fig — His-tagged SmVAL28 was recombinantly expressed in Exp293F cells, purified by affinity chromatography, and used in the splenic B cell assay, according to the legend to Fig 1A (n = 2). Intracellular IL-10 production (A) and CD86 GeoMFI (B) were assessed by flow cytometry. Representative FACS plots for intracellular IL-10 (C) and CD86 (D) expression of B cells for IL-10 FMO, PBS, SEA and SmVAL28 are shown. Secretion of IL-10 (E) and IL-6 (F) in supernatant were measured by ELISA. (TIF) [file pntd.0011344.s004.tif]

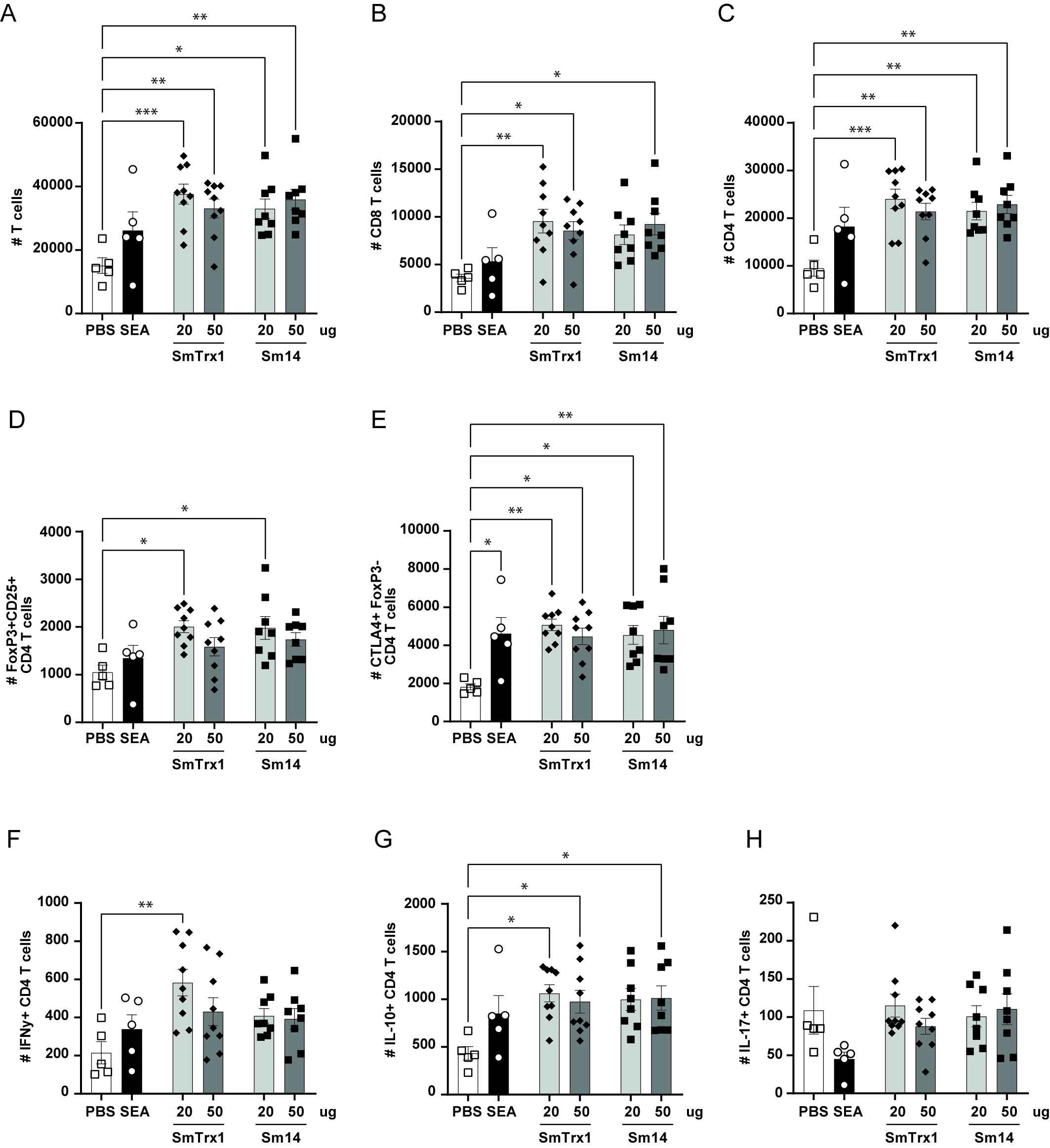

Supplement: S5 Fig — Mice were immunized s.c. into hock with PBS, 20 μg SEA, 20 or 50 μg SmTrx-1 or Sm14, and the draining popliteal LNs were analyzed 1 week later. Cells were counted, stimulated with PIB for 4 h and analyzed by flow cytometry. Figure shows the cell numbers of total T cells (A), CD4+ T cells (B), CD8+ T cells (C), FoxP3+CD25+ CD4 T cells (D), CTLA4+ CD4 T cells (E) as well as IFNγ, IL-10 and IL-17 producing CD4 T cells (F-H). n = 2. Error bars represent SEM, * p < 0.05, ** p < 0.01, *** p < 0.001, compared to PBS condition, with one-way ANOVA with Dunnett’s multiple comparisons test. (TIF) [file pntd.0011344.s005.tif]

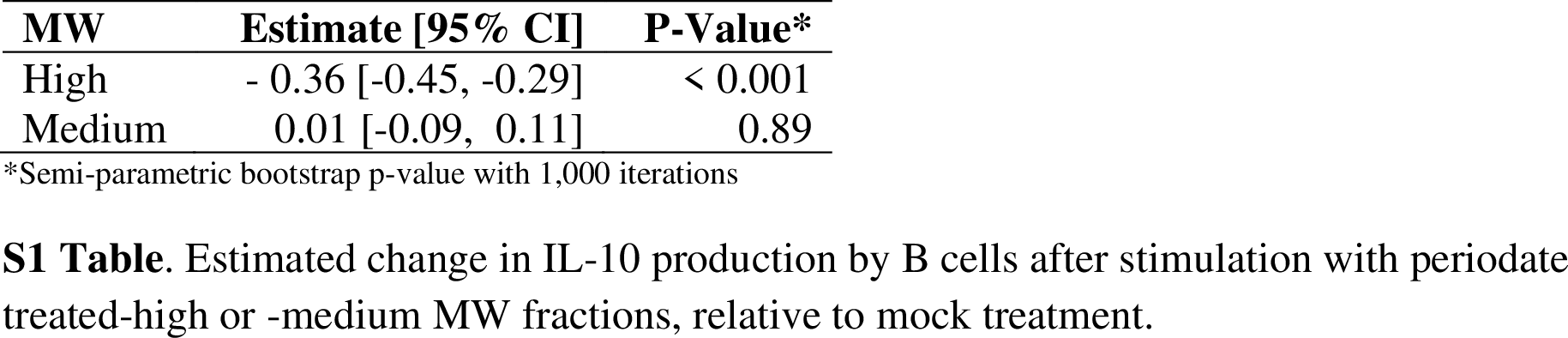

Supplement: S1 Table — (TIF) [file pntd.0011344.s006.tif]

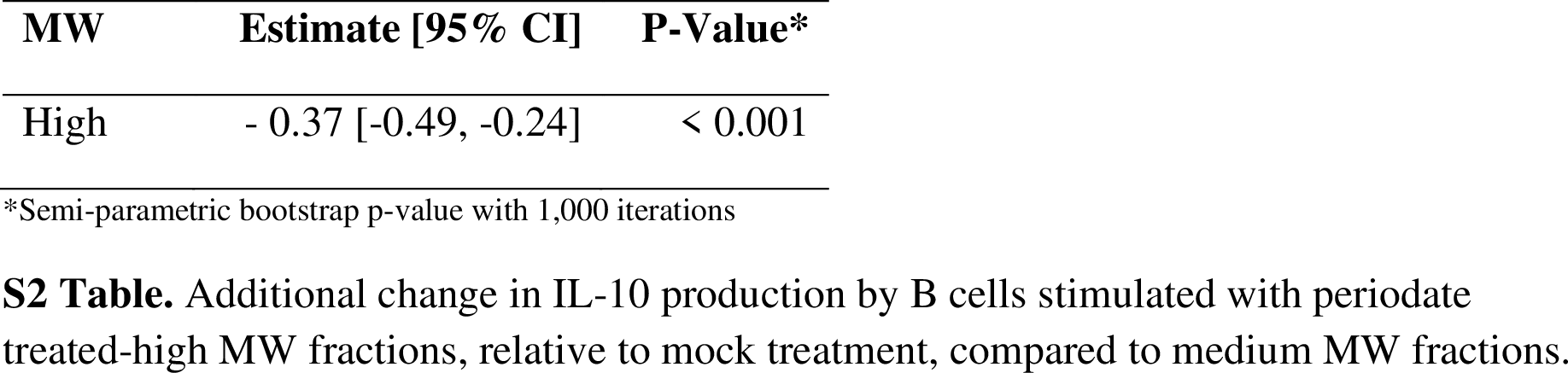

Supplement: S2 Table — (TIF) [file pntd.0011344.s007.tif]

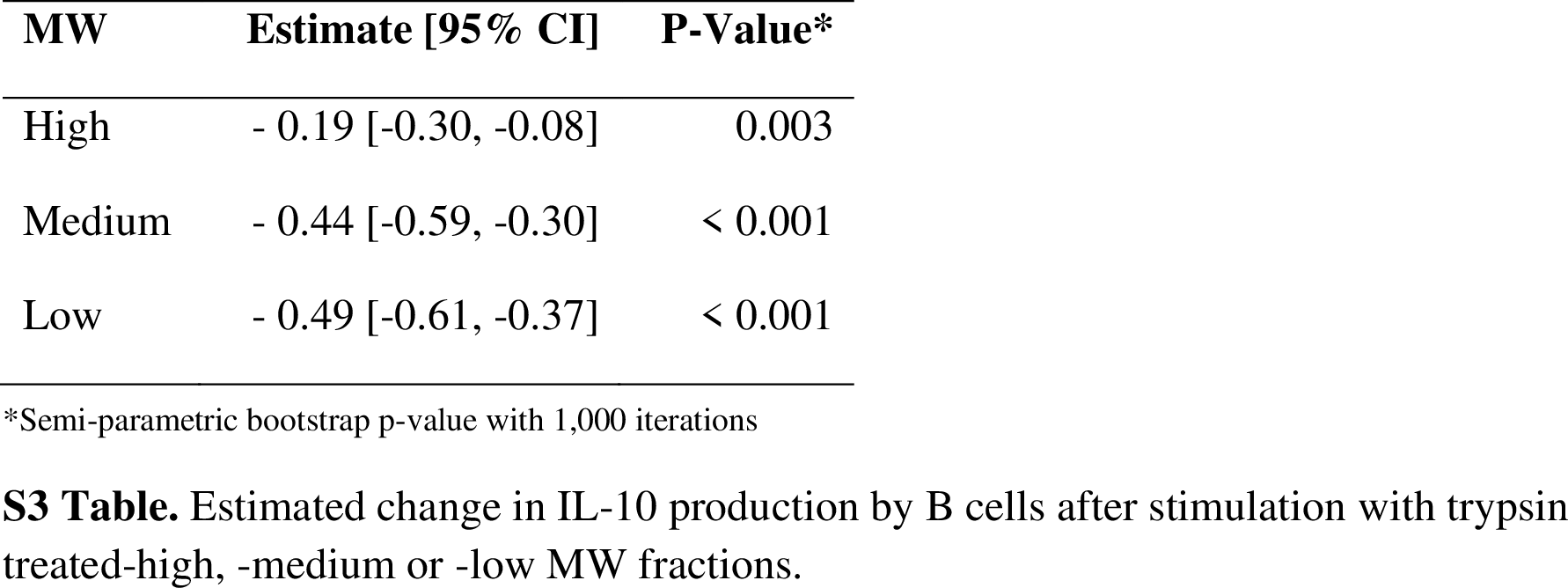

Supplement: S3 Table — (TIF) [file pntd.0011344.s008.tif]

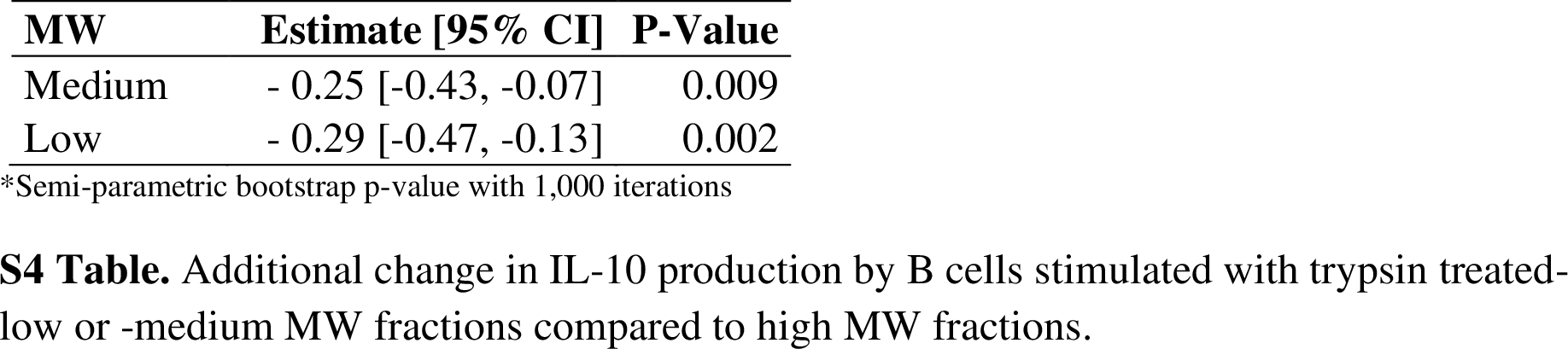

Supplement: S4 Table — (TIF) [file pntd.0011344.s009.tif]

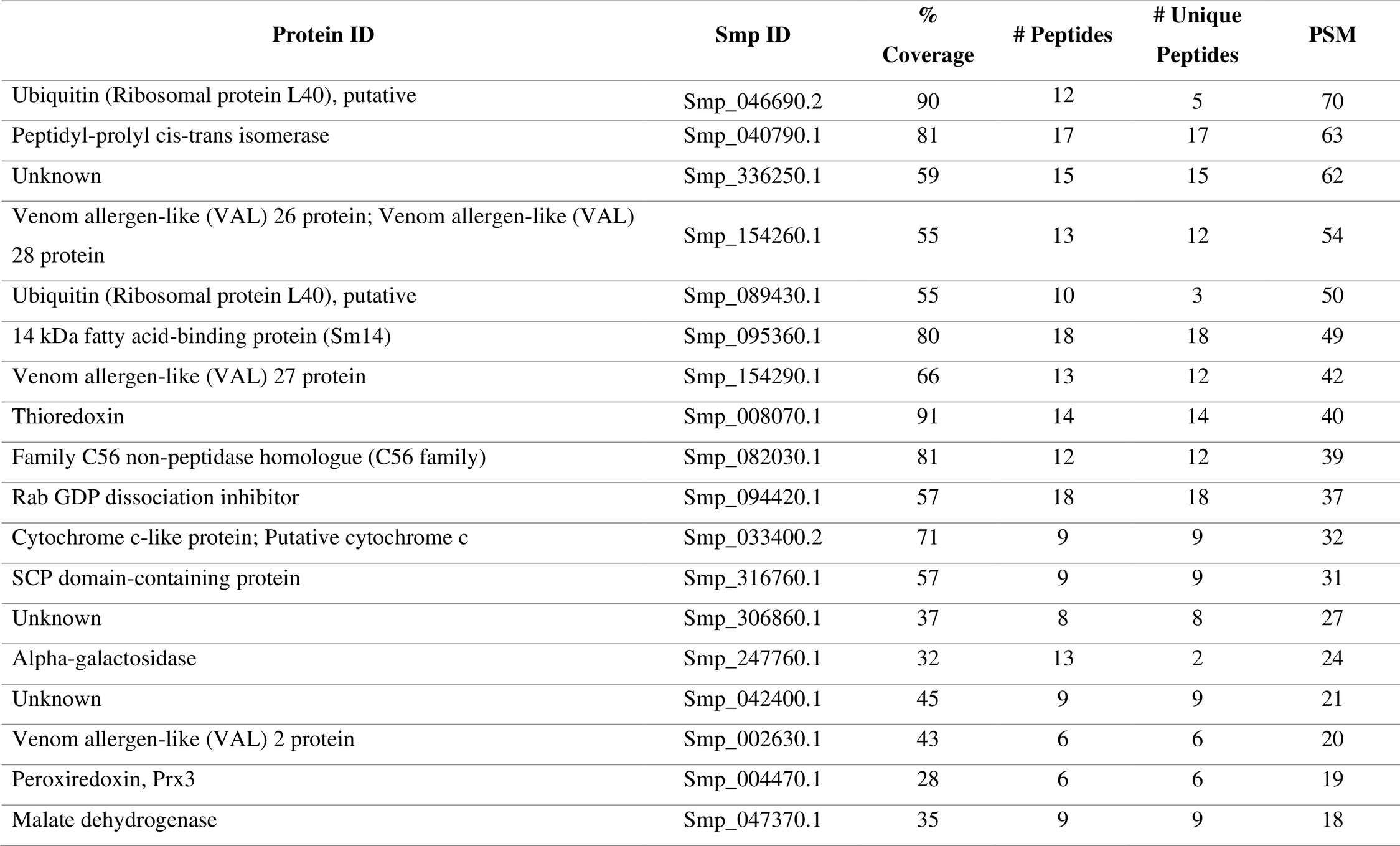

Supplement: S5 Table — (TIF) [file pntd.0011344.s010.tif]

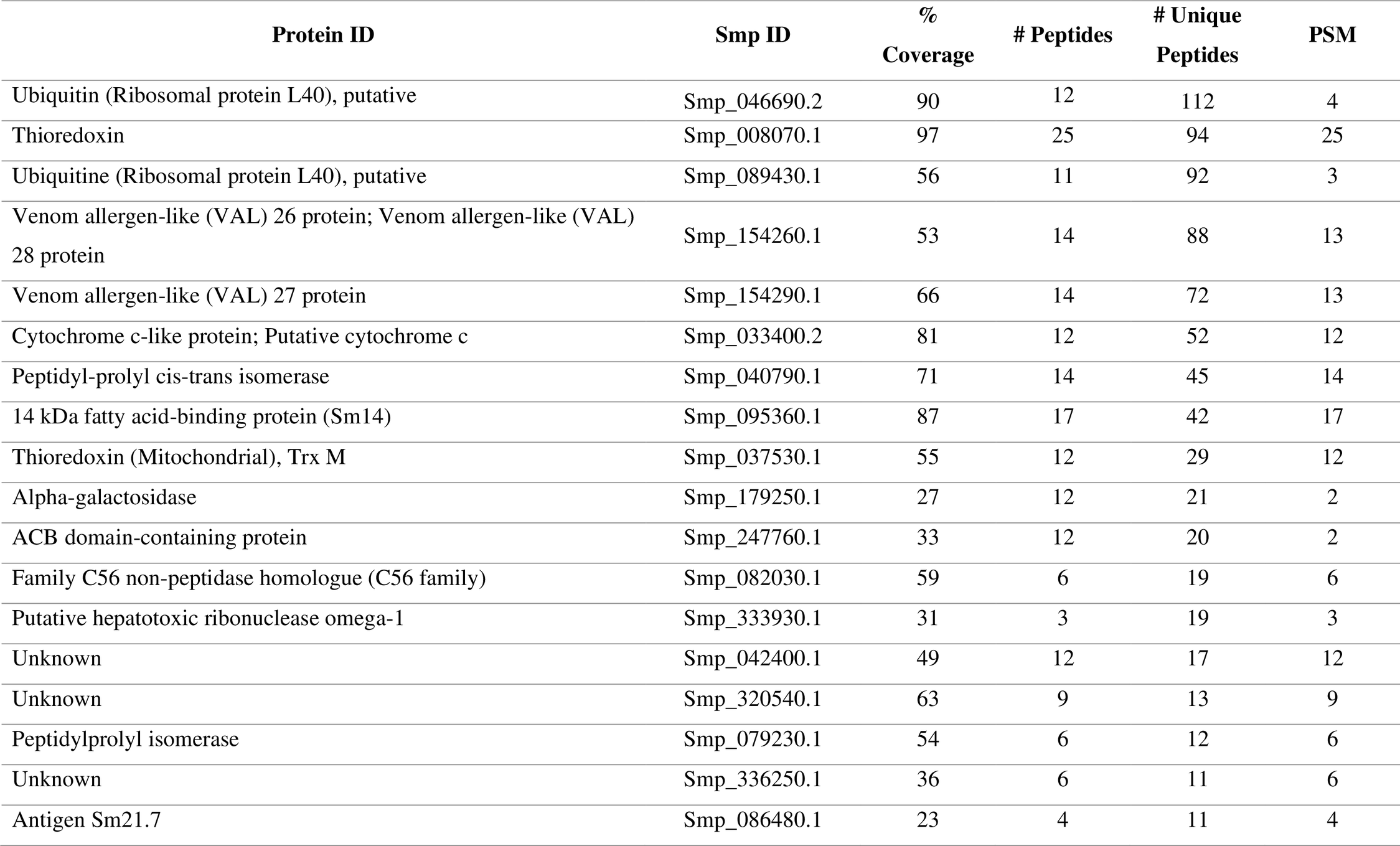

Supplement: S6 Table — (TIF) [file pntd.0011344.s011.tif]

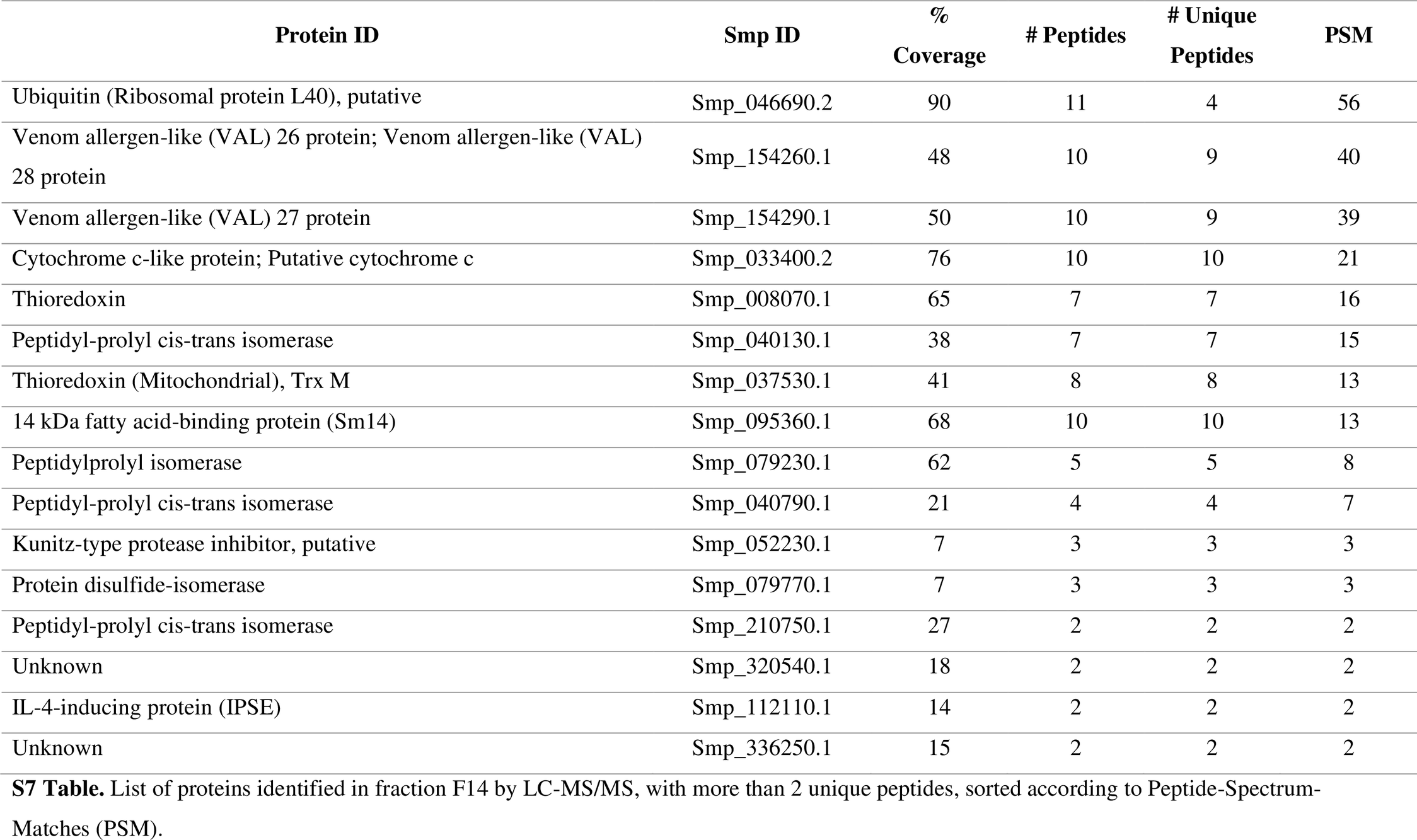

Supplement: S7 Table — (TIF) [file pntd.0011344.s012.tif]
